# Supplementary material for: Leveraging explainable artificial intelligence for early prediction of bloodstream infections using historical electronic health records
Source: PLOS Digit Health. 2024 Nov 14;3(11):e0000506. doi: 10.1371/journal.pdig.0000506 (PMC11563427; doi:10.1371/journal.pdig.0000506)
Supplement: S1 File — Additional materials and methods, including mathematical formulations for the ML models and performance metrics. (DOCX) [file pdig.0000506.s001.docx]

Advancing Bloodstream Infection Prediction Using Historical Electronic Health Records

(Supplementary Materials and Methods)

corresponding author: rajeev.bopche@ntnu.no

**1. SUPPLEMENTARY MATERIALS AND METHODS**

**1.1. Sequential ML Models**

For the purpose of this explanation, we define Y ={y_1_,y_2_,...,y_T_} as the input dataset consisting of medical events recorded over time *T*.

## **1.1.1. LSTM Model**

The LSTM (Long Short-Term Memory) model was designed with a sequential architecture incorporating two LSTM layers and two dense layers. The first LSTM layer contains 128 units and returns sequences, feeding into the second LSTM layer with 64 units. This sequential structure is effective in capturing temporal dependencies in the data. The dense layers, with 20 and 1 unit, respectively, use ReLU and sigmoid activations to introduce non-linearity and binary classification capability. The model is compiled using the Adam optimizer [7] with a learning rate of 0.0001, employing a binary cross-entropy loss function and accuracy as the performance metric. Class weights are utilized to address the issue of class imbalance [4]. The LSTM model was chosen for its superior ability to process sequential data, making it highly suitable for analyzing the time-dependent nature of patient medical records and laboratory results. Given the importance of temporal patterns in predicting BSIs, such as changes in laboratory markers over time or the timing of previous hospital admissions, LSTM's architecture enables the capture of long-term dependencies that might indicate the onset of an infection. The LSTM model is structured to handle sequences of data by remembering past information and using it to influence the current output, making it suitable for temporal data like medical histories. The model can be represented as follows:

Let *x_t_*_​_ be the input vector at time step *t*, and *h_t_*_​_ the hidden state vector, the LSTM updates are:

$$f_{t}= \sigma\left( W_{f}\cdot\left[ h_{t-1},x_{t} \right]+ b_{f} \right) \left( Forget gate \right)$$

$$i_{t}= \sigma\left( W_{i}\cdot\left[ h_{t-1},x_{t} \right]+ b_{i} \right) (Input gate)$$

$$o_{t}= \sigma\left( W_{o}\cdot\left[ h_{t-1},x_{t} \right]+ b_{o} \right) \left( Output gate \right)$$

$$\tilde{C_{t}}=\tanh\left( W_{C}\cdot\left[ h_{t-1},x_{t} \right]+ b_{C} \right)(Candidate memory cell)$$

$$C_{t}= f_{t} \odot C_{t-1}+i_{t} \odot\tilde{C_{t}} \left( Memory cell update \right)$$

$$h_{t}= o_{t} \odot tanh\left( C_{t} \right)(Hidden state update)$$

Where W and b are the weights and biases specific to each gate, σ is the sigmoid function, and ⊙ denotes element-wise multiplication.

### **1.1.2. GRU Model**

The Gated Recurrent Units (GRU) model, structured similarly to the LSTM model, comprises two GRU layers (128 and 64 units) followed by two dense layers. The GRU architecture, a variation of the standard LSTM, provides efficiency in modeling sequences with fewer parameters [3]. This model is compiled and trained under the same conditions as the LSTM model, targeting binary classifications. GRU offers a compelling balance between computational efficiency and the ability to model time-series data. Chosen for its reduced complexity while maintaining considerable performance in capturing temporal relationships, GRU was applied to evaluate its efficacy in BSI prediction under constraints of computational resources and training time. The GRU simplifies the LSTM architecture by combining the forget and input gates into a single "update gate" and merging the cell state and hidden state.

$$z_{t}= \sigma\left( W_{z}\cdot\left[ h_{t-1},x_{t} \right]+ b_{z} \right) (Update gate)$$

$$r_{t}= \sigma\left( W_{r}\cdot\left[ h_{t-1},x_{t} \right]+ b_{r} \right) \left( Reset gate \right)$$

$$\tilde{h_{t}}=\tanh\left( W\cdot\left[ r_{t} \odot h_{t-1},x_{t} \right]+ b \right)(Candidate hidden state)$$

$$h_{t}=\left( 1- z_{t} \right)\odot h_{t-1}+ z_{t} \odot\tilde{h_{t}}$$

**1.1.3. CNN-LSTM Model**

The CNN-LSTM model combines Convolutional Neural Network (CNN) and LSTM layers, capitalizing on the spatial feature extraction capabilities of CNNs [8] and the sequence modeling strength of LSTMs. The model starts with a CNN layer (64 filters, kernel size 3, ReLU activation), followed by MaxPooling to reduce dimensionality, an LSTM layer with 64 units for temporal data processing, and two dense layers. The CNN-LSTM model leverages the strengths of both CNNs in spatial feature extraction from sequential input data and LSTMs in understanding time-series data. This hybrid model was selected to exploit the spatial-temporal features present in patient records. The combination of CNNs for identifying significant features across a set of input data, followed by LSTM layers to interpret these features in a temporal context.

CNN layers extract spatial features:

$$F_{CNN}=ReLU(W_{conv} *x+b_{conv})$$

LSTM follows for temporal processing:

$$h_{t}, C_{t}=LSTM(F_{CNN},h_{t-1},C_{t-1})$$

**1.1.4. CNN-GRU Model**

Similar to the CNN-LSTM, the CNN-GRU model integrates CNN layers for spatial feature extraction with GRU layers for sequence modeling. The architecture includes a CNN layer, a MaxPooling layer for downsampling, followed by a GRU layer with 64 units, and two dense layers. The model is compiled and trained following the strategy applied to the LSTM and GRU models, making it suitable for datasets with both spatial and temporal characteristics.

CNN layers for feature extraction:

$$F_{CNN}=ReLU(W_{conv} *x+b_{conv})$$

GRU for sequence modeling:

$$h_{t}=GRU(F_{CNN},h_{t-1})$$

**1.1.5. Transformer Model**

This model employs the Transformer architecture [10], known for its effectiveness in handling sequential data with parallel processing and self-attention mechanisms. It includes two custom transformer blocks, each featuring multi-head attention, dropout for regularization, and feed-forward layers. The model concludes with global average pooling and dense layers for classification. Compiled with the Adam optimizer, it’s trained for 50 epochs to adapt to binary classification tasks, effectively addressing the complexities of sequential medical data. Transformer model’s self-attention mechanism allows for parallel processing of sequences, offering significant advantages in model training speed and the ability to focus on the most relevant parts of the patient's history for BSI prediction.

Self-attention and feed-forward networks are the primary components:

$$Attention\left( Q,K,V \right)=softmax (\frac{{QK}^{T}}{\surd d_{k}})V$$

Where, Q, K, V are the query, key, and value matrices derived from the input.

**1.1.6. DKN Model**

Densely Knowledge-aware Network (DKN) model is a sequential model that integrates domain knowledge into the prediction process [11]. The main components of the DKN model include:

***Embedding Layer:*** This layer transforms categorical features into dense vectors. Assume $x_{t}$represents the input at time step $t$. The embedding layer converts it into a vector $e_{t}$ as follows:

$$e_{t}=W_{embed}\cdot x_{t}$$

Where $W_{embed}$ ​ is the embedding matrix.

***Knowledge-aware Attention:*** This component applies an attention mechanism to combine the knowledge graph embeddings with the user embeddings. The attention score ​$\alpha_{t}$ ​ for the knowledge graph embedding $k_{t}$ at time $t$ is computed as:

$$\alpha_{t}=softmax(e_{t}\cdot W_{att}\cdot k_{t})$$

Where $W_{att}$ is a weight matrix, and $k_{t}$​ is the knowledge graph embedding.

***Dense Connection:*** The attention-weighted embeddings are passed through dense layers to create a sequence representation:

$$h_{t}=f(W_{dense}\cdot\left( \alpha_{t}\cdot e_{t} \right)+b_{dense})$$

Where $W_{dense}$​ and $b_{dense}$​ are the weight matrix and bias for the dense layer, and $f$ is the activation function (e.g., ReLU).

***Sequence Modeling***: The sequence of hidden states $h_{t}$​ ​ is then passed through an LSTM to capture temporal dependencies:

$$\vec{h}_{t}=LSTM(\vec{h}_{t-1},h_{t})$$

***Prediction Layer:*** Finally, the hidden states are passed through a sigmoid layer to produce the prediction $\hat{y}$ :

$$\hat{y}=\sigma(W_{out}\cdot\vec{h}_{T}+b_{out})$$

Where $W_{out}$ and ​$b_{out}$ are the output layer's weights and biases, and $\sigma$ is the sigmoid function.

**1.1.7. CapMatch Model**

The CapMatch model is a hybrid model that combines the Transformer architecture with capsule networks and contrastive learning [12].

*Transformer Encoder:* The model first uses a Transformer encoder to capture the sequential relationships. For an input sequence $\{x_{1},x_{2},\ldots,x_{T}\}$, the Transformer encodes the input as:

$$z_{t}=\mathrm{TransformerEncoder}(x_{t})=\mathrm{MultiAttention}(Q_{t},K_{t},V_{t})+\mathrm{FeedForward}(Q_{t},K_{t},V_{t})$$

Where $Q_{t},K_{t},V_{t}$ are the query, key, and value matrices at time step $t$.

*Capsule Network:* The output of the Transformer is then passed through a capsule network. Each capsule $C_{i}$ transforms the input $z_{t}$ into a higher-level representation:

$$C_{i}=\mathrm{Capsule}(z_{t})=\mathrm{Squash}(W_{caps}\cdot z_{t}+b_{caps})$$

Where $W_{caps}$​ and $b_{caps}$ are the capsule weights and biases, and the squash function ensures the output vector length is between 0 and 1.

*Contrastive Learning Objective:* The model uses a contrastive loss function to ensure that positive pairs (i.e., pairs from the same class) are closer in the embedding space than negative pairs. The contrastive loss $L_{contrastive}$​ for a pair $\left( z_{i},z_{j} \right)$is:

$$L_{contrastive}=\left( 1-y \right)\cdot{\max\left( 0,\mathrm{margin}-d\left( z_{i},z_{j} \right) \right)}^{2}+y\cdot{d\left( z_{i},z_{j} \right)}^{2}$$

Where $y=1$ if $z_{i}$ and $z_{j}$ are from the same class, and $y=0$ otherwise. The function $d\left( z_{i},z_{j} \right)$ measures the distance between the embeddings.

*Final Prediction:* The final prediction is made by passing the capsule outputs through a dense layer followed by a sigmoid activation:

$$\hat{y}=\sigma(W_{final}\cdot C_{i}+b_{final})$$

**1.2. Static ML Models:**

1.2.1. LightGBM Model: The LightGBM model, a gradient boosting framework [6], is utilized with the scale_pos_weight parameter adjusted to address class imbalances. This model was selected for its efficiency and speed in handling large-scale data. Its gradient boosting framework is capable of processing the extensive dataset with remarkable accuracy and minimal computational resources. LightGBM's use of histogram-based decision trees allows for faster training speed and lower memory usage, making it highly suitable for the complex task of BSI prediction.

LightGBM builds the model in a stage-wise fashion and generalize them by optimizing a loss function:

$$\hat{y}(x)= \sum_{k=1}^{K} f_{k}(x)$$

Where $f_{k}$ are the individual decision trees and$k$ is the number of boosting stages (trees).

1.2.2. CatBoost Model: CatBoost is another gradient boosting model [9] that is particularly effective in processing categorical features. Tuned for binary classification, it also focuses on handling class imbalance, a critical aspect in predicting BSIs.

CatBoost builds the model in a stage-wise fashion and generalize them by optimizing a loss function:

$$\hat{y}(x)= \sum_{k=1}^{K} f_{k}(x)$$

Where $f_{k}$ are the individual decision trees and$k$ is the number of boosting stages (trees).

1.2.3. XGBoost Model: The XGBoost (Extreme Gradient Boosting) model, stands out for its advanced regularization features [2] is employed with a focus on performance and speed. It uses the scale_pos_weight parameter for class imbalance and a logloss evaluation metric, making it suitable for binary classification.

XGBoost builds the model in a stage-wise fashion and generalize them by optimizing a loss function:

$$\hat{y}(x)= \sum_{k=1}^{K} f_{k}(x)$$

Where $f_{k}$ are the individual decision trees and$k$ is the number of boosting stages (trees).

1.2.4. ANN Model: A simple Artificial Neural Network (ANN) with three dense layers (128, 64, and 1 unit) is used, featuring ReLU activation for the first two layers and sigmoid activation for the output layer. This structure allows for modeling complex non-linear relationships in the data. The model is compiled using the Adam optimizer and binary cross-entropy loss. The ANN defined by the following equations, layer-wise:

$$z^{[l+1]}= W^{[l]} a^{[l]}+ b^{[l]}$$

$$a^{[l+1]}= g^{[l]} (z^{\left[ l+1 \right]})$$

Where $W^{[l]}$ and $b^{[l]}$are the weights and biases at layer $l$, $a^{[l]}$ is the activation from the previous layer, $g^{[l]}$

is the activation function, and $z^{\left[ l+1 \right]}$ is the linear combination input to the activation function at layer $l+1$.

1.2.5. RF Model: The Random Forest (RF) classifier [1] with 100 estimators is used for its robustness and effectiveness in handling high-dimensional data, making it suitable for complex medical datasets. A Random Forest aggregates the predictions of multiple decision trees, typically constructed with some form of randomness and then averaged to improve the predictive accuracy and control over-fitting. The prediction for a new sample $x$ is: $\hat{y}= \frac{1}{N} \sum_{i=1}^{N} t_{i}(x)$

Where $t_{i}$ are the individual decision trees and$N$ is the number of trees in the forest.

1.2.6. LR Model: A Logistic Regression (LR) model [5] is employed for binary classification, known for its simplicity and interpretability, which is crucial in medical settings where understanding model decisions is essential. The logistic function can be represented mathematically as:

$$P\left( y=1 | x \right)= \sigma(W^{T}x+b)$$

Where $\sigma\left( z \right)= \frac{1}{1+e^{-z}}$ is the logistic (sigmoid) function, $W$ is the weight vector, $x$ is the input feature vector, and b is the bias.

**1.3. Performance Metrics**

1.3.1. Accuracy:

This is the ratio of correctly predicted observations (both true positives and true negatives) to the total observations. It’s a general indicator of a model’s performance.

$$Accuracy=\frac{TP+TN}{TP+FP+FN+TN}$$

Where TP = True Positives, TN = True Negatives, FP = False Positives, FN = False Negatives.

1.3.2. Precision:

Also called Positive Predictive Value, it is the ratio of correctly predicted positive observations to the total predicted positive observations. It shows the model’s ability to return relevant results.

$$Precision= \frac{TP}{TP+FP}$$

1.3.3. Recall (Sensitivity or True Positive Rate):

This is the ratio of correctly predicted positive observations to all actual positives. It measures the model's capability to find all relevant cases.

​

$$Recall= \frac{TP}{TP+FN}$$

1.3.4. F1-score:

The F1-score is the harmonic mean of precision and recall, providing a balance between the two metrics. It’s especially useful when the class distribution is uneven.

​

$$F1-score=2 \times\frac{Precision \times Recall}{Precision+Recall}$$

1.3.5. Specificity (True Negative Rate):

This is the ratio of correctly predicted negative observations to all actual negatives. It measures the model's ability to identify negative results.

$$Specificity= \frac{TN}{TN+FP}$$

1.3.6. Area Under the Precision-Recall (PR) Curve (AUPRC):

This metric summarizes the trade-off between the true positive rate (recall) and the positive predictive value (precision) for a predictive model using different probability thresholds. The AUPRC is particularly insightful in the case of imbalanced datasets. The PR curve plots precision (y-axis) and recall (x-axis) for different threshold values, and AUPRC is the area under this curve.

1.3.7. Area Under the Receiver Operating Characteristic (AUROC):

This is used to evaluate the performance of a binary classification system by plotting the true positive rate (recall) against the false positive rate (1 - specificity) at various threshold settings. The AUROC is the area under the ROC curve, which ranges from 0 to 1. A model that predicts perfectly has an AUROC of 1, while a model that predicts randomly has an AUROC of 0.5. The ROC curve plots sensitivity (y-axis) versus 1-specificity (x-axis) for different threshold values, and AUROC is the area under this curve.

**1.4. Training Procedure**

The training of the ML models followed a structured and systematic process to ensure robustness and generalizability. The procedure incorporated several key steps.

1.4.1 Data Splitting:

The initial step involved splitting the dataset into training and testing subsets to validate the model’s performance. We used the **train_test_split** method from the **sklearn.model_selection** library, setting aside 20% of the data for testing. This split was performed without shuffling to preserve the temporal nature of the data. Further, the training data was divided into a smaller training set and a validation set, with 15% of the training data allocated for validation. This additional split allowed for the tuning of hyperparameters and the assessment of the model’s performance during training.

1.4.2. Data Scaling:

Given the sequential and tabular nature of the data, the necessary transformation steps were employed to prepare it for the corresponding learning algorithms. The data was reshaped and scaled using the StandardScaler from sklearn.preprocessing. This normalization step is crucial for models that are sensitive to the scale of input features. After scaling, the data was reshaped back to its original form, ensuring compatibility with the machine learning models used.

1.4.3. Anomaly Detection:

An Isolation Forest model was implemented to detect anomalies within the data. This model was trained exclusively on normal data (non-infection cases) and then used to compute anomaly scores for the training, validation, and test sets. These scores were normalized and appended to the original dataset to serve as additional features, enhancing the model’s ability to distinguish between normal and anomalous patterns

References

1. Breiman, L. (2001). Random forests. Machine learning, 45(1), 5-32.
2. Chen, T., & Guestrin, C. (2016, August). XGBoost: A scalable tree boosting system. In Proceedings of the 22nd ACM SIGKDD international conference on Knowledge discovery and data mining (pp. 785-794).
3. Cho, K., Van Merriënboer, B., Bahdanau, D., & Bengio, Y. (2014). On the properties of neural machine translation: Encoder-decoder approaches. arXiv preprint arXiv:1409.1259.
4. He, H., & Garcia, E. A. (2009). Learning from imbalanced data. IEEE Transactions on Knowledge and Data Engineering, 21(9), 1263-1284.
5. Hosmer Jr, D. W., Lemeshow, S., & Sturdivant, R. X. (2013). Applied logistic regression (Vol. 398). John Wiley & Sons.
6. Ke, G., Meng, Q., Finley, T., Wang, T., Chen, W., Ma, W., ... & Liu, T. Y. (2017). Lightgbm: A highly efficient gradient boosting decision tree. Advances in neural information processing systems, 30.
7. Kingma, D. P., & Ba, J. (2014). Adam: A method for stochastic optimization. arXiv preprint arXiv:1412.6980.
8. LeCun, Y., Bengio, Y., & Hinton, G. (1998). Gradient-based learning applied to document recognition. Proceedings of the IEEE, 86(11), 2278-2324.
9. Prokhorenkova, L., Gusev, G., Vorobev, A., Dorogush, A. V., & Gulin, A. (2018). Catboost: unbiased boosting with categorical features. Advances in neural information processing systems, 31.
10. Vaswani, A., Shazeer, N., Parmar, N., Uszkoreit, J., Jones, L., Gomez, A. N., ... & Polosukhin, I. (2017). Attention is all you need. Advances in neural information processing systems, 30.
11. Hongwei Wang, Fuzheng Zhang, Xing Xie, and Minyi Guo. 2018. DKN: Deep Knowledge-Aware Network for News Recommendation. In Proceedings of the 2018 World Wide Web Conference (WWW '18). International World Wide Web Conferences Steering Committee, Republic and Canton of Geneva, CHE, 1835–1844. https://doi.org/10.1145/3178876.3186175
12. Z. Xiao *et al*., "CapMatch: Semi-Supervised Contrastive Transformer Capsule With Feature-Based Knowledge Distillation for Human Activity Recognition," in *IEEE Transactions on Neural Networks and Learning Systems*, doi: 10.1109/TNNLS.2023.3344294.
