# Supplementary material for: Leveraging explainable artificial intelligence for early prediction of bloodstream infections using historical electronic health records
Source: PLOS Digit Health. 2024 Nov 14;3(11):e0000506. doi: 10.1371/journal.pdig.0000506 (PMC11563427; doi:10.1371/journal.pdig.0000506)
Supplement: S2 File — Contains additional results on statistical analysis and description of predictors. Fig A: Correlation matrix of clinical predictors Table A. Comparison of the predictors (Top 25 most influential) between the two classes. Table B. Statistical significance of clinical Predictors. Table C. Correlation coefficients of most correlated Features. Table D. List of all the predictors, their description and their average values across the dataset. Table E. List of ICD-10 codes used to classify selected diseases. Table F. List of microbes identified as contaminants List A. Groups of various microbiology tests. (DOCX) [file pdig.0000506.s002.docx]

Advancing Bloodstream Infection Prediction Using Historical Electronic Health Records

(Supplementary Results)

**2. SUPPLEMENTARY RESULTS**

**2.1. Correlation Matrix**

The correlation matrix analysis given in Fig A. provide deeper insights into the complex relationships between clinical features. For example, Bilirubin levels, showed significant correlations with ICD_K, which is concerned with liver functions. The strong positive correlation between ICD_N and creatinine suggests an association between kidney function and creatinine levels.





**Fig A. Correlation Matrix Analysis of Clinical Features for BSI Prediction:** This heatmap provides a visual representation of the strength and directionality of correlations between various clinical features within the EPRs, highlighting the complex interdependencies relevant to BSI prediction.

**2.2. Statistical Analysis**

Table A lists the features extracted from EPRs with their mean values for positive and negative BSI cases, along with the T-statistic and p-value, demonstrating their statistical significance in distinguishing between the two groups.

For comprehensive details on the ICD-10 codes used in this study, please refer to the Norwegian Directorate of eHealth's ICD-10 code directory: [https://finnkode.ehelse.no/#icd10/0/0/0/-1](For%20comprehensive%20details%20on%20the%20ICD-10%20codes%20used%20in%20this%20study,%20please%20refer%20to%20the%20Norwegian%20Directorate%20of%20eHealth's%20ICD-10%20code%20directory:%20https:/finnkode.ehelse.no#icd10/0/0/0/-1    Additional information on clinical procedures relevant to our methodology can be found at the Norwegian Clinical Procedure Code Directory (NKPK): https://www.ehelse.no/kodeverk-og-terminologi/Norsk-klinisk-prosedyrekodeverk-(NKPK))

Additional information on clinical procedures relevant to our methodology can be found at the Norwegian Clinical Procedure Code Directory (NKPK): [https://www.ehelse.no/kodeverk-og-terminologi/Norsk-klinisk-prosedyrekodeverk-(NKPK)](For%20comprehensive%20details%20on%20the%20ICD-10%20codes%20used%20in%20this%20study,%20please%20refer%20to%20the%20Norwegian%20Directorate%20of%20eHealth's%20ICD-10%20code%20directory:%20https:/finnkode.ehelse.no#icd10/0/0/0/-1    Additional information on clinical procedures relevant to our methodology can be found at the Norwegian Clinical Procedure Code Directory (NKPK): https://www.ehelse.no/kodeverk-og-terminologi/Norsk-klinisk-prosedyrekodeverk-(NKPK))

**Table A.** Comparison of the predictors (Top 25 most influential) between the two classes.

| Comparison of the predictors (Top 25 most influential) between the two classes | | | | |
| --- | --- | --- | --- | --- |
| Feature names | Class 1 (mean)  **(n=5288)** | Class 0 (mean) **(n=60687)** | T-Statistics | P-Value |
| ICD_A (infectious diseases) | 0.344175492 | 0.127045331 | -41.35067292 | 0 |
| ICD_B (infectious diseases) | 0.270990923 | 0.098044062 | -34.86262822 | 6.9733E-264 |
| ICD_N (kidney disease) | 0.498108926 | 0.255112298 | -31.06397543 | 2.4511E-210 |
| BILIRUBIN TOTAL (total bilirubin) | 14.64849131 | 7.316773443 | -23.83455936 | 6.0583E-125 |
| prior_positive_blood_culture_test | 0.370083207 | 0.178852143 | -20.33423563 | 1.22442E-91 |
| ICD_J (influenza, pneumonia etc.) | 0.307677761 | 0.488885593 | 16.95136079 | 2.5746E-64 |
| ICD_R (abnormal symptoms and findings) | 0.298411498 | 0.188771895 | -16.06835215 | 5.48158E-58 |
| Age (at the time of BC) | 68.87821483 | 65.07332707 | -14.51708417 | 1.11892E-47 |
| KREATININE (creatinine) | 99.42019885 | 82.73778714 | -13.30695014 | 2.42534E-40 |
| CRP | 110.8463237 | 94.62243471 | -12.20533489 | 3.21434E-34 |
| ICD_A_aggregate (infectious diseases) | 0.797655068 | 0.557236311 | -11.29534135 | 1.47299E-29 |
| LEUKOCYTTER (leukocytes) | 11.57262042 | 10.27756163 | -10.07214858 | 7.68314E-24 |
| procedure_G_aggregate (procedures on Chest wall, pleura, diaphragm, trachea, bronchi, lungs and mediastinum) | 0.996596067 | 1.48710597 | 9.453454788 | 3.3812E-21 |
| ICD_U (unsure etiology and antimicrobial resistance) | 0.018910741 | 0.006953713 | -9.34670256 | 9.3135E-21 |
| ICD_J_aggregate (influenza, pneumonia etc) | 1.876134644 | 2.99597937 | 9.271600994 | 1.88697E-20 |
| procedure_K (procedures on the ears) | 0.073940998 | 0.038377247 | -8.468324845 | 2.5398E-17 |
| TROMBOCYTTER (thrombocytes) | 156.5213799 | 174.202004 | 8.409629616 | 4.20742E-17 |
| ICD_K (gastrointestinal diseases) | 0.234871407 | 0.179527741 | -8.148452108 | 3.75005E-16 |
| procedure_J (procedures on endocrine glands) | 0.184379728 | 0.127407847 | -6.851217934 | 7.38635E-12 |
| prior_positive_edta (ethylenediaminetetraacetic acid) | 0.142965204 | 0.254074184 | 6.204949673 | 5.50384E-10 |
| ICD_M (musculoskeletal disease) | 0.067133132 | 0.092490978 | 5.438316649 | 5.39768E-08 |
| ICD_B_aggregate (infectious diseases) | 0.752458396 | 0.554468008 | -5.194454856 | 2.06E-07 |
| ICD_R_aggregate (abnormal symptoms and findings) | 2.291603631 | 2.686555605 | 5.06204369 | 4.15899E-07 |
| Explicit_sepsis (sepsis episode) | 0.006807867 | 0.002751825 | -5.034421936 | 4.80552E-07 |
| LOS (length of stay of most recent medical episode) | 8.751426185 | 8.013890262 | -4.578791274 | 4.68523E-06 |

**Table B. Statistical Significance of Clinical Features in BSI Prediction**

| Feature | Mean_1 | Mean_0 | T-Stat | P-Value |
| --- | --- | --- | --- | --- |
| ICD_A (INfectious diseases) | 0,344175492 | 0,127045331 | -41,35067292 | 0 |
| ICD_B (infectious diseases) | 0,270990923 | 0,098044062 | -34,86262822 | 6,9733E-264 |
| ICD_N (Kidney disease) | 0,498108926 | 0,255112298 | -31,06397543 | 2,4511E-210 |
| BILIRUBIN TOTAL | 14,64849131 | 7,316773443 | -23,83455936 | 6,0583E-125 |
| prior_positive_blood_culture_test | 0,370083207 | 0,178852143 | -20,33423563 | 1,22442E-91 |
| ICD_J (INfluenza, pneumonia etc) | 0,307677761 | 0,488885593 | 16,95136079 | 2,5746E-64 |
| ICD_R (abnormal symptoms and findings) | 0,298411498 | 0,188771895 | -16,06835215 | 5,48158E-58 |
| age | 68,87821483 | 65,07332707 | -14,51708417 | 1,11892E-47 |
| CREATININe | 99,42019885 | 82,73778714 | -13,30695014 | 2,42534E-40 |
| CRP | 110,8463237 | 94,62243471 | -12,20533489 | 3,21434E-34 |
| ICD_A_aggregate | 0,797655068 | 0,557236311 | -11,29534135 | 1,47299E-29 |
| LEUKOCYTEs | 11,57262042 | 10,27756163 | -10,07214858 | 7,68314E-24 |
| procedure_G_aggregate | 0,996596067 | 1,48710597 | 9,453454788 | 3,3812E-21 |
| ICD_U (unsure etiology and antimicrobial resistance) | 0,018910741 | 0,006953713 | -9,34670256 | 9,3135E-21 |
| ICD_J_aggregate | 1,876134644 | 2,99597937 | 9,271600994 | 1,88697E-20 |
| procedure_K | 0,073940998 | 0,038377247 | -8,468324845 | 2,5398E-17 |
| THROMBOCYTES | 156,5213799 | 174,202004 | 8,409629616 | 4,20742E-17 |
| ICD_K (Gastrointestinal diseases) | 0,234871407 | 0,179527741 | -8,148452108 | 3,75005E-16 |
| procedure_J | 0,184379728 | 0,127407847 | -6,851217934 | 7,38635E-12 |
| prior_positive_edta | 0,142965204 | 0,254074184 | 6,204949673 | 5,50384E-10 |
| ICD_M (muscelo sceletal disease) | 0,067133132 | 0,092490978 | 5,438316649 | 5,39768E-08 |
| ICD_B_aggregate | 0,752458396 | 0,554468008 | -5,194454856 | 2,06E-07 |
| ICD_R_aggregate | 2,291603631 | 2,686555605 | 5,06204369 | 4,15899E-07 |
| explicitsepsis | 0,006807867 | 0,002751825 | -5,034421936 | 4,80552E-07 |
| LenGTH OF STAY | 8,751426185 | 8,013890262 | -4,578791274 | 4,68523E-06 |
| lung | 0,004538578 | 0,011600508 | 4,484655057 | 7,31549E-06 |
| prior_positive_urin | 1,929462935 | 1,694909948 | -4,408070386 | 1,0446E-05 |
| urgency_code | 1,534795764 | 1,608449915 | 4,362127709 | 1,28999E-05 |
| procedure_G | 0,118759455 | 0,150641818 | 4,281756985 | 1,85685E-05 |
| procedure_P_aggregate | 0,40714826 | 0,486133768 | 4,280229403 | 1,86964E-05 |
| procedure_H_aggregate | 0,014561271 | 0,029577999 | 4,147999385 | 3,35813E-05 |
| BILIRUBIN conjugated | 0,232311321 | 0,065033406 | -4,11215013 | 3,92544E-05 |
| dementia | 0,023638427 | 0,01593422 | -4,099000172 | 4,15435E-05 |
| prior_positive_bronki | 0,02269289 | 0,034834479 | 4,093154034 | 4,26051E-05 |
| prior_positive_naso | 0,098524962 | 0,123238914 | 3,849162537 | 0,000118634 |
| urinary tract infection | 0,064485628 | 0,05207046 | -3,842260912 | 0,00012202 |
| procedure_J_aggregate | 1,404122542 | 1,210852407 | -3,828843833 | 0,000128866 |
| procedure_M_aggregate | 0,135590015 | 0,21706461 | 3,554880295 | 0,000378415 |
| infection | 0,382942511 | 0,35323875 | -3,526113532 | 0,000421992 |
| ICD_O_aggregate (Pregnancy and childbirth) | 0,121028744 | 0,205694795 | 3,462795609 | 0,000534933 |
| Gender | 0,577534039 | 0,55371002 | -3,344127893 | 0,000825876 |
| LACTATE Patient close analysis (PNA) | 0,008075022 | 0,003636033 | -3,323379816 | 0,000889901 |
| ICD_G_aggregate (DiseSES in nerve system) | 1,103441755 | 1,409033236 | 3,283993066 | 0,001024007 |
| procedure_N_aggregate | 0,878593041 | 0,989388172 | 3,222718706 | 0,001270418 |
| prior_positive_hal | 0,077912254 | 0,096577521 | 3,187923318 | 0,001433654 |
| procedure_F_aggregate | 1,45688351 | 1,614283125 | 2,957700927 | 0,003100525 |
| cancer | 0,115355522 | 0,101965825 | -2,947414542 | 0,003205555 |
| procedure_5_aggregate | 0,009266263 | 0,005025788 | -2,904486047 | 0,003679787 |
| prior_positive_melk | 0,031580938 | 0,022591329 | -2,884077265 | 0,003926894 |
| time_to_last | 2972,138616 | 2703,685155 | -2,830881686 | 0,004643392 |
| pneumonia | 0,013237519 | 0,018669567 | 2,829259213 | 0,004666997 |
| ICD_S_aggregate (injuries, poisoning) | 0,67984115 | 0,786066209 | 2,80971113 | 0,004960057 |
| ICD_L (skin and subcutanous tissue) | 0,036308623 | 0,045314483 | 2,806499098 | 0,005009773 |
| procedure_A_aggregate | 0,689107413 | 0,773575889 | 2,714061928 | 0,0066481 |
| procedure_I_aggregate | 1,089069592 | 1,549755302 | 2,709280601 | 0,006744669 |
| procedure_X_aggregate | 0,038956127 | 0,048066307 | 2,593785484 | 0,009494666 |
| PO2 patient close analysis (PNA) | 0,049101527 | 0,027645026 | -2,571385896 | 0,010131766 |
| hsCRP- high-sensitivity | 0,435938155 | 0,220883538 | -2,562084132 | 0,010407174 |
| prior_positive_blod | 0,000945537 | 0,000280126 | -2,541905562 | 0,011027263 |
| ICD_Z (contact with health care) | 0,208396369 | 0,192116928 | -2,508297178 | 0,012133839 |
| ICD_S | 0,031959153 | 0,043600771 | 2,417076551 | 0,01564842 |
| procedure_4_aggregate | 0,085098336 | 0,142617035 | 2,398584708 | 0,016461328 |
| procedure_N | 0,062594554 | 0,081549591 | 2,394110082 | 0,016663522 |
| procedure_B_aggregate | 0,0153177 | 0,02488177 | 2,393410542 | 0,016695328 |
| procedure_7_aggregate | 0,002080182 | 0,000708554 | -2,343323555 | 0,019115753 |
| ICD_P | 0,000378215 | 6,5912E-05 | -2,284147895 | 0,022366002 |
| ICD_K_aggregate | 2,448373676 | 2,6887307 | 2,282838148 | 0,02244307 |
| ICD_E_aggregate | 2,228630862 | 2,503995913 | 2,271078931 | 0,023145408 |
| ICD_M_aggregate | 2,531202723 | 2,903389523 | 2,228408258 | 0,025856659 |
| procedure_L_aggregate | 0,239599092 | 0,277687149 | 2,181580206 | 0,029144046 |
| procedure_Z_aggregate | 2,7346823 | 3,162390627 | 2,164119749 | 0,030458736 |
| ICD_U_aggregate | 0,101739788 | 0,048033351 | -2,128036049 | 0,033337791 |
| endocarditis | 0,000567322 | 0,001829057 | 2,118814976 | 0,034109833 |
| procedure_R | 0,101172466 | 0,091205695 | -2,058743528 | 0,039522731 |
| procedure_A | 0,024773071 | 0,032659383 | 2,048298666 | 0,040534693 |
| PH Patient close analysis (PNA) | 0,034281222 | 0,021877451 | -2,021893797 | 0,043192017 |
| care_level_code | 1,384833585 | 1,420436008 | 1,990852146 | 0,046501262 |
| procedure_F | 0,087178517 | 0,100598151 | 1,976468227 | 0,048105929 |
| ICD_F_aggregate (psychiatry) | 2,827723147 | 3,633364642 | 1,954044491 | 0,050700175 |
| ICU_LOS | 2,981706998 | 2,650340964 | -1,932316255 | 0,053327581 |
| procedure_X | 0,001512859 | 0,002966039 | 1,891273562 | 0,058592212 |
| ICD_T | 0,06505295 | 0,072569084 | 1,843891203 | 0,065203479 |
| ICD_L_aggregate | 1,180597579 | 1,346466294 | 1,706002883 | 0,08801225 |
| procedure_E_aggregate | 0,074697428 | 0,086839026 | 1,686321207 | 0,091738677 |
| procedure_D | 0,006429652 | 0,00911233 | 1,554480206 | 0,120074711 |
| prior_positive_TOUNGE | 0,003025719 | 0,00476214 | 1,552239142 | 0,120609811 |
| ICD_G | 0,069024206 | 0,075304431 | 1,526496124 | 0,126891149 |
| procedure_P | 0,03801059 | 0,043831463 | 1,480473498 | 0,138751704 |
| procedure_W_aggregate | 4,665468986 | 4,917445252 | 1,448242424 | 0,147554016 |
| ICD_C_aggregate | 10,1327534 | 10,70029825 | 1,44494589 | 0,148477834 |
| procedure_D_aggregate | 0,761157337 | 0,827491885 | 1,384282469 | 0,166276662 |
| ICD_E | 0,233736762 | 0,223194424 | -1,375344331 | 0,169029389 |
| skin and soft tissue infection | 0,028555219 | 0,03186844 | 1,319573214 | 0,18698213 |
| prior_positive_eye | 0,005673222 | 0,007513965 | 1,29696494 | 0,194647786 |
| prior_positive_ear | 0,007186082 | 0,010051576 | 1,292375505 | 0,196231662 |
| ICD_Q_aggregate | 0,181164902 | 0,234350026 | 1,255203297 | 0,209409415 |
| ICD_H | 0,011913767 | 0,014220509 | 1,245365277 | 0,213001934 |
| ICD_I | 0,53214826 | 0,54985417 | 1,235580109 | 0,216619082 |
| sepsis | 0,007186082 | 0,005882644 | -1,178385412 | 0,238647241 |
| ICD_F | 0,084341906 | 0,089788587 | 1,166514471 | 0,243410718 |
| ICD_D | 0,131807867 | 0,138695272 | 1,166308364 | 0,243494009 |
| ICD_D_aggregate | 1,658472012 | 1,754263681 | 1,130222716 | 0,258386499 |
| procedure_O_aggregate | 2,393343419 | 2,575592796 | 1,104059726 | 0,269571257 |
| procedure_O | 0,003593041 | 0,006096858 | 1,05934796 | 0,289445219 |
| prior_positive_JOINT | 0,004160363 | 0,002949561 | -0,995856519 | 0,319323517 |
| ICD_W | 0,002836611 | 0,003691071 | 0,991906525 | 0,321246764 |
| LACTATE | 0,002283618 | 0,001299838 | -0,989945226 | 0,322205121 |
| procedure_E | 0,003214826 | 0,004547926 | 0,985089253 | 0,324583844 |
| ICD_Y | 0,0153177 | 0,013643779 | -0,984101745 | 0,325069098 |
| total_los | 42,87793904 | 43,61798037 | 0,870115801 | 0,384240289 |
| ICD_Z_aggregate | 10,17719365 | 10,74928733 | 0,852094677 | 0,394164639 |
| ICD_T_aggregate | 0,929273828 | 0,882330647 | -0,835738649 | 0,403304974 |
| procedure_C | 0,001891074 | 0,002966039 | 0,833749271 | 0,404425306 |
| ICU_LOS_total | 4,442593757 | 4,279581362 | -0,822741344 | 0,410660035 |
| prior_positive_plasma | 0,303517398 | 0,29106728 | -0,798046402 | 0,424846436 |
| procedure_Q | 0,030068079 | 0,033351459 | 0,796967893 | 0,425472547 |
| procedure_B | 0,000189107 | 0,00041195 | 0,783043327 | 0,43360448 |
| ICD_V_aggregate | 0,0102118 | 0,008189563 | -0,717607838 | 0,473001665 |
| ICD_I_aggregate | 5,705748865 | 5,840493022 | 0,717445016 | 0,473102092 |
| prior_positive_OTHER | 0,030446293 | 0,032659383 | 0,694628612 | 0,487290523 |
| procedure_H | 0,000567322 | 0,000856856 | 0,624606892 | 0,532231255 |
| procedure_L | 0,006996974 | 0,006179248 | -0,567866095 | 0,570127828 |
| ICD_P_aggregate | 0,000567322 | 0,000378994 | -0,547356784 | 0,584135491 |
| prior_positive_faeces | 0,031959153 | 0,033763409 | 0,528405193 | 0,597219912 |
| ICD_Q | 0,010022693 | 0,009293588 | -0,476530918 | 0,633697764 |
| procedure_T_aggregate | 0,999621785 | 1,019097995 | 0,459568441 | 0,64582753 |
| ICD_Y_aggregate | 0,078857791 | 0,076474368 | -0,457219624 | 0,6475147 |
| LACTATE BLOOD GAS VENOUS | 0 | 4,98218E-05 | 0,455463913 | 0,64877727 |
| ICD_X | 0,001134644 | 0,001367673 | 0,442787861 | 0,657920636 |
| intra abdominal infection | 0,000189107 | 0,000296604 | 0,441842589 | 0,658604566 |
| procedure_Y_aggregate | 0,002269289 | 0,002718869 | 0,431397388 | 0,666180859 |
| procedure_U | 0,03876702 | 0,037438002 | -0,420602699 | 0,674046593 |
| LACTATE BLODGASS | 0 | 1,9801E-05 | 0,407792515 | 0,683427551 |
| procedure_Z | 0,041603631 | 0,042974607 | 0,406800962 | 0,6841555 |
| cardiovascular | 0,284228442 | 0,281559477 | -0,3820645 | 0,702414754 |
| organ dysfunction | 0,10381997 | 0,105574505 | 0,377813673 | 0,705570231 |
| ICD_V | 0,000567322 | 0,000708554 | 0,373156925 | 0,709032852 |
| prior_positive_skin | 0,031013616 | 0,029874602 | -0,365892023 | 0,714446822 |
| procedure_C_aggregate | 0,763426626 | 0,782309226 | 0,363956238 | 0,715891855 |
| procedure_Y | 0,001891074 | 0,002208051 | 0,343233039 | 0,731424245 |
| prior_positive_bONE | 0,000189107 | 0,000131824 | -0,342077887 | 0,732293368 |
| ICD_C | 0,335098336 | 0,331603144 | -0,335447843 | 0,737288349 |
| central nervous system | 0,000378215 | 0,000296604 | -0,32695392 | 0,743703775 |
| procedure_M | 0,007753404 | 0,007134971 | -0,325191736 | 0,745036994 |
| procedure_Q_aggregate | 0,992435703 | 0,968477598 | -0,322642792 | 0,746966805 |
| procedure_T | 0,03801059 | 0,037059008 | -0,302524468 | 0,762253238 |
| PO2 | 0 | 6,06804E-05 | 0,292076803 | 0,770228972 |
| procedure_K_aggregate | 3,791792738 | 3,949626773 | 0,285532633 | 0,775237003 |
| procedure_W | 0,128782148 | 0,127160677 | -0,284648114 | 0,775914639 |
| ICD_W_aggregate | 0,041792738 | 0,04271096 | 0,275024497 | 0,783298279 |
| ICD_O | 0,014561271 | 0,015406924 | 0,266777934 | 0,789640979 |
| BILIRUBIN UNCONJUGATED | 0,036051213 | 0,031755005 | -0,25897492 | 0,795655533 |
| PH | 0,011321129 | 0,010442195 | -0,24493993 | 0,806503831 |
| prior_positive_biopsY | 0,001512859 | 0,001384151 | -0,230712383 | 0,817538968 |
| ICD_N_aggregate | 6,423411498 | 6,290441116 | -0,211106351 | 0,832804936 |
| procedure_R_aggregate | 1,252269289 | 1,224430273 | -0,19640446 | 0,84429421 |
| procedure_I | 0,002269289 | 0,002405787 | 0,115573469 | 0,907990942 |
| procedure_U_aggregate | 1,198751891 | 1,200833786 | 0,062239456 | 0,950372331 |
| ICD_H_aggregate | 1,618570348 | 1,613805263 | -0,055689755 | 0,95558913 |
| prior_positive_anus | 0,021558245 | 0,021454348 | -0,038644183 | 0,969174192 |
| ICD_X_aggregate | 0,048411498 | 0,048346433 | -0,004004121 | 0,996805194 |

Detailed top three most correlated coefficients among significant clinical features identified in the EPRs are provided in Table B.

**Table C. Correlation Coefficients of Most Correlated Features**

| Feature | Most Correlated | Second Most | Third Most |
| --- | --- | --- | --- |
| ICD_A | ICD_A_aggregate (0.336) | LOS (0.162) | procedure_R (0.156) |
| ICD_B | ICD_B_aggregate (0.223) | ICD_N (0.213) | urgency_code (0.109) |
| ICD_N | KREATININ (0.340) | ICD_B (0.213) | prior_positive_urin (0.203) |
| BILIRUBIN TOTAL | BILIRUBIN KONJUGERT (0.252) | ICD_K (0.204) | ICD_K_aggregate (0.099) |
| prior_positive_blood_culture_test | ICD_A_aggregate (0.286) | prior_positive_urin (0.283) | infection (0.220) |
| ICD_J | ICD_J_aggregate (0.362) | procedure_G (0.346) | procedure_G_aggregate (0.273) |
| ICD_R | ICD_R_aggregate (0.151) | ICD_N (0.110) | urgency_code (0.099) |
| age | procedure_M_aggregate (0.239) | ICD_O_aggregate (0.213) | KREATININ (0.176) |
| KREATININ | ICD_N (0.340) | age (0.176) | Gender (0.143) |
| CRP | LEUKOCYTTER (0.223) | LOS (0.098) | KREATININ (0.095) |
| ICD_A_aggregate | ICD_A (0.336) | prior_positive_blood_culture_test (0.286) | prior_positive_naso (0.242) |
| LEUKOCYTTER | CRP (0.223) | TROMBOCYTTER (0.200) | urgency_code (0.119) |
| procedure_G_aggregate | ICD_J_aggregate (0.524) | prior_positive_edta (0.388) | prior_positive_bronki (0.294) |
| ICD_U | ICD_U_aggregate (0.162) | prior_positive_urin (0.132) | prior_positive_blood_culture_test (0.109) |
| ICD_J_aggregate | procedure_G_aggregate (0.524) | prior_positive_edta (0.464) | ICD_J (0.362) |
| procedure_K | ICD_N (0.190) | LOS (0.130) | procedure_J (0.082) |
| TROMBOCYTTER | LEUKOCYTTER (0.200) | urgency_code (0.143) | ICD_J (0.105) |
| ICD_K | procedure_J (0.350) | ICD_K_aggregate (0.261) | BILIRUBIN TOTAL (0.204) |
| procedure_J | ICD_K (0.350) | LOS (0.347) | procedure_J_aggregate (0.272) |
| prior_positive_edta | ICD_J_aggregate (0.464) | procedure_G_aggregate (0.388) | lung (0.328) |
| ICD_M | ICD_M_aggregate (0.184) | procedure_N (0.157) | procedure_N_aggregate (0.136) |
| ICD_B_aggregate | ICD_B (0.223) | prior_positive_urin (0.161) | prior_positive_blood_culture_test (0.144) |
| ICD_R_aggregate | ICD_K_aggregate (0.371) | prior_positive_urin (0.301) | procedure_A_aggregate (0.273) |
| explicitsepsis | prior_positive_blood_culture_test (0.171) | urgency_code (0.077) | care_level_code (0.064) |
| LOS | procedure_J (0.347) | procedure_G (0.275) | procedure_R (0.248) |
| lung | prior_positive_edta (0.328) | ICD_J_aggregate (0.228) | procedure_G_aggregate (0.205) |
| prior_positive_urin | infection (0.468) | urinarytractinfection (0.373) | ICD_R_aggregate (0.301) |
| urgency_code | care_level_code (0.598) | ICD_J (0.227) | time_to_last (0.187) |
| procedure_G | ICD_J (0.346) | LOS (0.275) | procedure_G_aggregate (0.252) |
| procedure_P_aggregate | procedure_G_aggregate (0.196) | ICD_A_aggregate (0.180) | ICD_E_aggregate (0.159) |
| procedure_H_aggregate | Gender (0.117) | procedure_P_aggregate (0.068) | procedure_L_aggregate (0.053) |
| BILIRUBIN KONJUGERT | BILIRUBIN TOTAL (0.252) | ICD_K (0.040) | ICD_K_aggregate (0.025) |
| dementia | age (0.118) | infection (0.069) | urinarytractinfection (0.050) |
| prior_positive_bronki | procedure_G_aggregate (0.294) | prior_positive_edta (0.271) | ICD_J_aggregate (0.181) |
| prior_positive_naso | prior_positive_hal (0.287) | ICD_A_aggregate (0.242) | ICD_J_aggregate (0.160) |
| urinarytractinfection | infection (0.459) | prior_positive_urin (0.373) | prior_positive_blood_culture_test (0.182) |
| procedure_J_aggregate | ICD_K_aggregate (0.384) | procedure_A_aggregate (0.329) | procedure_J (0.272) |
| procedure_M_aggregate | ICD_O_aggregate (0.779) | age (0.239) | procedure_L_aggregate (0.214) |
| infection | prior_positive_urin (0.468) | urinarytractinfection (0.459) | ICD_R_aggregate (0.231) |
| ICD_O_aggregate | procedure_M_aggregate (0.779) | procedure_L_aggregate (0.235) | age (0.213) |
| Gender | procedure_L_aggregate (0.252) | procedure_M_aggregate (0.147) | KREATININ (0.143) |
| LAKTAT PNA | PH PNA (0.791) | PO2 PNA (0.758) | ICD_J (0.024) |
| ICD_G_aggregate | procedure_A_aggregate (0.284) | ICD_R_aggregate (0.209) | procedure_Z_aggregate (0.158) |
| procedure_N_aggregate | procedure_N (0.329) | ICD_M_aggregate (0.321) | ICD_S_aggregate (0.223) |
| prior_positive_hal | prior_positive_naso (0.287) | ICD_A_aggregate (0.230) | prior_positive_blood_culture_test (0.160) |
| procedure_F_aggregate | procedure_G_aggregate (0.254) | procedure_F (0.232) | procedure_X_aggregate (0.201) |
| cancer | Gender (0.128) | age (0.102) | procedure_J_aggregate (0.095) |
| prior_positive_melk | procedure_J_aggregate (0.186) | ICD_K_aggregate (0.096) | prior_positive_blood_culture_test (0.080) |
| time_to_last | urgency_code (0.187) | infection (0.117) | care_level_code (0.106) |
| pneumonia | infection (0.217) | prior_positive_edta (0.216) | ICD_J_aggregate (0.174) |
| ICD_S_aggregate | procedure_N_aggregate (0.223) | ICD_S (0.182) | ICD_R_aggregate (0.145) |
| ICD_L | ICD_B (0.049) | ICD_J (0.047) | ICD_M (0.040) |
| procedure_A_aggregate | procedure_J_aggregate (0.329) | ICD_G_aggregate (0.284) | ICD_R_aggregate (0.273) |
| procedure_I_aggregate | procedure_Z_aggregate (0.634) | ICD_S_aggregate (0.142) | age (0.121) |
| procedure_X_aggregate | procedure_F_aggregate (0.201) | procedure_G_aggregate (0.103) | procedure_A_aggregate (0.073) |
| PO2 PNA | PH PNA (0.942) | LAKTAT PNA (0.758) | TROMBOCYTTER (0.029) |
| prior_positive_blod | prior_positive_blood_culture_test (0.120) | ICD_A_aggregate (0.116) | prior_positive_urin (0.073) |
| ICD_Z | procedure_F_aggregate (0.196) | time_to_last (0.085) | procedure_F (0.078) |
| ICD_S | procedure_N (0.266) | ICD_S_aggregate (0.182) | procedure_A (0.104) |
| procedure_N | procedure_N_aggregate (0.329) | ICD_S (0.266) | ICD_M (0.157) |
| procedure_B_aggregate | ICD_E_aggregate (0.227) | ICD_K_aggregate (0.069) | procedure_P_aggregate (0.059) |
| ICD_P | ICD_G_aggregate (0.103) | prior_positive_naso (0.061) | procedure_G (0.019) |
| ICD_K_aggregate | procedure_J_aggregate (0.384) | ICD_R_aggregate (0.371) | ICD_K (0.261) |
| ICD_E_aggregate | procedure_B_aggregate (0.227) | prior_positive_urin (0.208) | prior_positive_edta (0.185) |
| procedure_L_aggregate | Gender (0.252) | ICD_O_aggregate (0.235) | procedure_M_aggregate (0.214) |
| procedure_Z_aggregate | procedure_I_aggregate (0.634) | ICD_M_aggregate (0.351) | ICD_J_aggregate (0.209) |
| ICD_U_aggregate | procedure_5_aggregate (0.164) | ICD_U (0.162) | ICD_A_aggregate (0.151) |
| endocarditis | infection (0.076) | procedure_F_aggregate (0.060) | prior_positive_blood_culture_test (0.050) |
| procedure_R | LOS (0.248) | ICD_A (0.156) | procedure_N (0.139) |
| procedure_A | procedure_A_aggregate (0.155) | ICD_S (0.104) | procedure_G (0.091) |
| PH PNA | PO2 PNA (0.942) | LAKTAT PNA (0.791) | TROMBOCYTTER (0.032) |
| care_level_code | urgency_code (0.598) | LOS (0.240) | ICD_J (0.192) |
| procedure_F | procedure_F_aggregate (0.232) | procedure_G (0.129) | LOS (0.120) |

**Table D.** List of all the features, description and their average values across the dataset.

| Feature name | Mean across the dataset | Feature Description |
| --- | --- | --- |
| blood_culture_test_encoded | 0.08015157256536568 | Encoded results of blood culture tests |
| urgency_code | 1.6025464190981433 | Urgency code of the current hospital episode |
| care_level_code | 1.4175824175824177 | Care level code for the current hospital episode |
| LENGTH OF STAY (LOS) | 8.07300492610854 | Length of stay of current episode till the BC test |
| time_to_last_EPISODE | 2725.202122015915 | Time distance to the most recent episode |
| total_los | 43.558664898320835 | Total cumulative LOS per patient |
| age | 65.37829480863964 | Age at the time of BC |
| procedure_A | 0.03202728306176582 | Count of Procedure codes starting with the letter in the recent episode |
| procedure_B | 0.00039408866995073894 | Count of Procedure codes starting with the letter |
| procedure_C | 0.0028798787419477075 | Count of Procedure codes starting with the letter |
| procedure_D | 0.008897309586964759 | Count of Procedure codes starting with the letter |
| procedure_E | 0.004441076165214096 | Count of Procedure codes starting with the letter |
| procedure_F | 0.09952254641909815 | Count of Procedure codes starting with the letter |
| procedure_G | 0.14808639636225843 | Count of Procedure codes starting with the letter |
| procedure_H | 0.0008336491095111784 | Count of Procedure codes starting with the letter |
| procedure_I | 0.0023948465327775672 | Count of Procedure codes starting with the letter |
| procedure_J | 0.13197423266388783 | Count of Procedure codes starting with the letter |
| procedure_K | 0.04122773777946192 | Count of Procedure codes starting with the letter |
| procedure_L | 0.006244789693065555 | Count of Procedure codes starting with the letter |
| procedure_M | 0.007184539598332702 | Count of Procedure codes starting with the letter |
| procedure_N | 0.08003031451307313 | Count of Procedure codes starting with the letter |
| procedure_O | 0.005896172792724517 | Count of Procedure codes starting with the letter |
| procedure_P | 0.04336491095111785 | Count of Procedure codes starting with the letter |
| procedure_Q | 0.033088291019325505 | Count of Procedure codes starting with the letter |
| procedure_R | 0.09200454717696097 | Count of Procedure codes starting with the letter |
| procedure_T | 0.03713527851458886 | Count of Procedure codes starting with the letter |
| procedure_U | 0.037544524441076164 | Count of Procedure codes starting with the letter |
| procedure_W | 0.12729064039408866 | Count of Procedure codes starting with the letter |
| procedure_X | 0.0028495642288745736 | Count of Procedure codes starting with the letter |
| procedure_Y | 0.002182644941265631 | Count of Procedure codes starting with the letter |
| procedure_Z | 0.04286472148541114 | Count of Procedure codes starting with the letter |
| ICD_A | 0.14444865479348237 | Count of ICD codes starting with the letter ‘A’ in the recent episode |
| ICD_B | 0.11190602500947329 | Count of ICD codes starting with the letter ‘B’ |
| ICD_C | 0.3318832891246684 | Count of ICD codes starting with the letter ‘C’ |
| ICD_D | 0.13814323607427056 | Count of ICD codes starting with the letter ‘D’ |
| ICD_E | 0.22403940886699508 | Count of ICD codes starting with the letter ‘E’ |
| ICD_F | 0.08935202728306177 | Count of ICD codes starting with the letter ‘F’ |
| ICD_G | 0.07480106100795755 | Count of ICD codes starting with the letter ‘G’ |
| ICD_H | 0.014035619552860932 | Count of ICD codes starting with the letter ‘H’ |
| ICD_I | 0.5484350132625995 | Count of ICD codes starting with the letter ‘I’ |
| ICD_J | 0.4743615005683971 | Count of ICD codes starting with the letter ‘J’ |
| ICD_K | 0.18396362258431223 | Count of ICD codes starting with the letter ‘K’ |
| ICD_L | 0.04459264873057976 | Count of ICD codes starting with the letter ‘L’ |
| ICD_M | 0.09045850701023114 | Count of ICD codes starting with the letter ‘M’ |
| ICD_N | 0.2745888594164456 | Count of ICD codes starting with the letter ‘N’ |
| ICD_O | 0.015339143615005685 | Count of ICD codes starting with the letter ‘O’ |
| ICD_P | 9.09435392194013e-05 | Count of ICD codes starting with the letter ‘P’ |
| ICD_Q | 0.009352027283061766 | Count of ICD codes starting with the letter ‘Q’ |
| ICD_R | 0.19755968169761273 | Count of ICD codes starting with the letter ‘R’ |
| ICD_S | 0.04266767715043577 | Count of ICD codes starting with the letter ‘S’ |
| ICD_T | 0.07196665403561955 | Count of ICD codes starting with the letter ‘T’ |
| ICD_U | 0.007912087912087912 | Count of ICD codes starting with the letter ‘U’ |
| ICD_V | 0.0006972338006820765 | Count of ICD codes starting with the letter ‘V’ |
| ICD_W | 0.0036225843122394846 | Count of ICD codes starting with the letter ‘W’ |
| ICD_X | 0.0013489958317544524 | Count of ICD codes starting with the letter ‘X’ |
| ICD_Y | 0.013777946191739296 | Count of ICD codes starting with the letter ‘Y’ |
| ICD_Z | 0.19342175066312997 | Count of ICD codes starting with the letter ‘Z’ |
| urinary tract infection | 0.05306555513452065 | Count of urinary tract infection episodes |
| cardiovascular | 0.2817733990147783 | Count of cardivascular episodes |
| lung | 0.011034482758620689 | Count of lung disease episodes |
| central nervous system | 0.00030314513073133765 | Count of CNS disease episodes |
| organ dysfunction | 0.10543387646835922 | Count of organ dysfunction episodes |
| skin and soft tissue infection | 0.03160287987874195 | Count of skin and soft tissue infection episodes |
| pneumonia | 0.01823417961348996 | Count of pheumonia episodes |
| endocarditis | 0.0017279272451686245 | Count of endocarditis episodes |
| sepsis | 0.005987116331943918 | Count of sepsis episodes |
| infection | 0.35561955286093216 | Count of infection episodes |
| dementia | 0.016551724137931035 | Count of dementia episodes |
| explicit sepsis | 0.003076923076923077 | Count of explicit sepsis episodes |
| intra abdominal infection | 0.00028798787419477074 | Count of Intra abdominal infection episodes |
| cancer | 0.10303902993558166 | Count of cancer episodes |
| ICD_A_aggregate | 0.5765062523683213 | Total number of the codes in the history |
| ICD_B_aggregate | 0.5703372489579386 | Total number of the codes in the history |
| ICD_C_aggregate | 10.654808639636226 | Total number of the codes in the history |
| ICD_D_aggregate | 1.7465858279651383 | Total number of the codes in the history |
| ICD_E_aggregate | 2.481924971580144 | Total number of the codes in the history |
| ICD_F_aggregate | 3.568791208791209 | Total number of the codes in the history |
| ICD_G_aggregate | 1.3845395983327018 | Total number of the codes in the history |
| ICD_H_aggregate | 1.6141871921182267 | Total number of the codes in the history |
| ICD_I_aggregate | 5.8296930655551344 | Total number of the codes in the history |
| ICD_J_aggregate | 2.9062220538082606 | Total number of the codes in the history |
| ICD_K_aggregate | 2.669465706707086 | Total number of the codes in the history |
| ICD_L_aggregate | 1.3331716559302766 | Total number of the codes in the history |
| ICD_M_aggregate | 2.873558165971959 | Total number of the codes in the history |
| ICD_N_aggregate | 6.301098901098901 | Total number of the codes in the history |
| ICD_O_aggregate | 0.1989086775293672 | Total number of the codes in the history |
| ICD_P_aggregate | 0.00039408866995073894 | Total number of the codes in the history |
| ICD_Q_aggregate | 0.23008715422508527 | Total number of the codes in the history |
| ICD_R_aggregate | 2.654899583175445 | Total number of the codes in the history |
| ICD_S_aggregate | 0.7775521030693444 | Total number of the codes in the history |
| ICD_T_aggregate | 0.8860932171276998 | Total number of the codes in the history |
| ICD_U_aggregate | 0.05233800682076544 | Total number of the codes in the history |
| ICD_V_aggregate | 0.008351648351648353 | Total number of the codes in the history |
| ICD_W_aggregate | 0.04263736263736264 | Total number of the codes in the history |
| ICD_X_aggregate | 0.04835164835164835 | Total number of the codes in the history |
| ICD_Y_aggregate | 0.07666540356195528 | Total number of the codes in the history |
| ICD_Z_aggregate | 10.703433118605533 | Total number of the codes in the history |
| procedure_0_aggregate | 0.9127396741189845 | Total number of the codes in the history |
| procedure_1_aggregate | 0.29888594164456234 | Total number of the codes in the history |
| procedure_2_aggregate | 0.011792345585449034 | Total number of the codes in the history |
| procedure_3_aggregate | 0.08447139067828723 | Total number of the codes in the history |
| procedure_4_aggregate | 0.13800682076544146 | Total number of the codes in the history |
| procedure_5_aggregate | 0.005365668813944676 | Total number of the codes in the history |
| procedure_6_aggregate | 0.006002273588480485 | Total number of the codes in the history |
| procedure_7_aggregate | 0.0008184918529746116 | Total number of the codes in the history |
| procedure_9_aggregate | 0.003849943160287988 | Total number of the codes in the history |
| procedure_A_aggregate | 0.7668056081849185 | Total number of the codes in the history |
| procedure_B_aggregate | 0.024115195149677908 | Total number of the codes in the history |
| procedure_C_aggregate | 0.7807957559681697 | Total number of the codes in the history |
| procedure_D_aggregate | 0.8221750663129973 | Total number of the codes in the history |
| procedure_E_aggregate | 0.08586585827965139 | Total number of the codes in the history |
| procedure_F_aggregate | 1.6016672982190223 | Total number of the codes in the history |
| procedure_G_aggregate | 1.4477908298597955 | Total number of the codes in the history |
| procedure_H_aggregate | 0.028374384236453203 | Total number of the codes in the history |
| procedure_I_aggregate | 1.5128306176582038 | Total number of the codes in the history |
| procedure_J_aggregate | 1.2263433118605533 | Total number of the codes in the history |
| procedure_K_aggregate | 3.9369761273209547 | Total number of the codes in the history |
| procedure_L_aggregate | 0.27463433118605535 | Total number of the codes in the history |
| procedure_M_aggregate | 0.21053429329291398 | Total number of the codes in the history |
| procedure_N_aggregate | 0.980507768093975 | Total number of the codes in the history |
| procedure_O_aggregate | 2.5609852216748767 | Total number of the codes in the history |
| procedure_P_aggregate | 0.4798029556650246 | Total number of the codes in the history |
| procedure_Q_aggregate | 0.9703978779840848 | Total number of the codes in the history |
| procedure_R_aggregate | 1.226661614247821 | Total number of the codes in the history |
| procedure_T_aggregate | 1.0175369458128078 | Total number of the codes in the history |
| procedure_U_aggregate | 1.200666919287609 | Total number of the codes in the history |
| procedure_W_aggregate | 4.897248957938613 | Total number of the codes in the history |
| procedure_X_aggregate | 0.047336112163698374 | Total number of the codes in the history |
| procedure_Y_aggregate | 0.002682834406972338 | Total number of the codes in the history |
| procedure_Z_aggregate | 3.1281091322470633 | Total number of the codes in the history |
| Gender | 0.5556195528609321 | Gender |
| Intensive Care UNIT (ICU)_LOS | 2.67687025621688 | LOS of current or recent ICU admission |
| ICU_LOS_total | 4.292632196921903 | Total cumulative LOS in ICUs |
| BILIRUBIN KONJUGERT | 0.07818219805313276 | Test results from most recent test |
| BILIRUBIN TOTAL | 7.893079244009708 | Test results from most recent test |
| BILIRUBIN UKONJUGERT | 0.032092705721718046 | Test results from most recent test |
| CRP | 95.89770491738955 | Test results from most recent test |
| CRP-HØYSENSITIV | 0.2377877926480454 | Test results from most recent test |
| CREATININE | 84.0490992796357 | Test results from most recent test |
| LACTATE | 0.0013771672728556804 | Test results from most recent test |
| LACTATE BLODGASS | 1.8244523700224818e-05 | Test results from most recent test |
| LACTATE BLODGASS VENØST | 4.590557576185599e-05 | Test results from most recent test |
| LACTATE PNA | 0.003984957095942651 | Test results from most recent test |
| LEUKOCYTEs | 10.379359038494 | Test results from most recent test |
| PH | 0.010511283158931385 | Test results from most recent test |
| PH PNA | 0.022852443000576792 | Test results from most recent test |
| PO2 | 5.591063714585025e-05 | Test results from most recent test |
| PO2 PNA | 0.029331603046246928 | Test results from most recent test |
| THROMBOCYTES | 172.81222789456044 | Test results from most recent test |
| prior_positive_OTHER | 0.032482000757862825 | Total number of positives for other microbiology tests |
| prior_positive_anus | 0.021462675255778704 | Total number of positives for particular microbiology tests |
| prior_positive_bONE | 0.00013641530882910194 | Total number of positives for particular microbiology tests |
| prior_positive_biopsY | 0.001394467601364153 | Total number of positives for particular microbiology tests |
| prior_positive_bloOd | 0.0003334596438044714 | Total number of positives for particular microbiology tests |
| prior_positive_blood_culture_test | 0.19417961348995832 | Total number of positives for particular microbiology tests |
| prior_positive_bronki | 0.03386131110269041 | Total number of positives for particular microbiology tests |
| prior_positive_melk | 0.023311860553239864 | Total number of positives for particular microbiology tests |
| prior_positive_edta | 0.2451686244789693 | Total number of positives for particular microbiology tests |
| prior_positive_faeces | 0.03361879499810534 | Total number of positives for particular microbiology tests |
| prior_positive_hal | 0.09508147025388404 | Total number of positives for particular microbiology tests |
| prior_positive_skin | 0.029965896172792723 | Total number of positives for particular microbiology tests |
| prior_positive_joint | 0.003046608563849943 | Total number of positives for particular microbiology tests |
| prior_positive_nasophARYNX | 0.12125805229253506 | Total number of positives for particular microbiology tests |
| prior_positive_plasma | 0.29206517620310724 | Total number of positives for particular microbiology tests |
| prior_positive_tOUNGE | 0.004622963243652899 | Total number of positives for particular microbiology tests |
| prior_positive_urinE | 1.7137097385373248 | Total number of positives for particular microbiology tests |
| prior_positive_ear | 0.009821902235695339 | Total number of positives for particular microbiology tests |
| prior_positive_eye | 0.007366426676771504 | Total number of positives for particular microbiology tests |

**Table E. Disease Groups:** List of ICD-10 codes used to classify selected diseases.

| **S/No.** | **Disease** | **ICD-10 codes** |
| --- | --- | --- |
| **1** | **Explicit Sepsis** | 'A021','A207','A217','A227','A241','A267','A282','A327','A394','A40','A41','A427','B007','B377' |
| **2** | **Organ dysfunction** | 'D695','E872','G934','I46','I959','J80','J952','J96','K720','K729','N00', 'N17','R090','R092','R400','R401','R402','R41', 'R55', 'R57', 'R651', 'R572' |
| **3** | **Implicit Sepsis** | Organ dysfunction + Infection |
| **4** | **Infection** | 'A00','A01','A02','A03','A04','A05','A06','A07','A08','A09','A19', 'A20','A21','A22','A23','A24','A25','A26','A27','A28','A30','A31','A32','A36', 'A37','A38','A39','A42', 'A43', 'A44', 'A46','A48','A49','A54','A59','A690','A691','A699','A70','A74', 'A75', 'A77','A78','A79','A80','A81','A83','A84','A85','A86','A87', 'A88','A89','A90','A91','A92','A93','A94','A95','A96','A97','A98', 'A99','B00','B01','B02','B03','B04','B05','B06','B08','B09','B10','B25', 'B26','B27','B33','B34','B37','B38','B39','B40','B41','B42','B43','B44', 'B45','B46','B48','B49','B50','B54','B55','B57','B58','B59','B60','B64', 'B67','B95','B96','B97','B99','G00','G01','G02','G03','G04','G05','G06', 'G07','G08','H050', 'H602','H700','I00','I33','I38','I39','I400','J01','J02','J03',  'J04','J05','J06','J09','J10','J11','J12','J13','J14','J15','J16','J17','J18','J19','J20',  'J21','J22','J36','J390','J391','J85','J86','K35','K36','K37','K61','K630',   'K631','K65','K750', 'K810', 'K830','L02','L03','L030','L04','L08','M00','M01','M86','N10','N151', 'N30','N390','N410','N412','N413','N45','N70','N71','N72','N73','N74', 'N980','O030','O035','O045','O080','O23','O753', 'O85',  'O86','O883','O91','O98','R02','T802','T814','T826''T827','T835', 'T836','T845','T846','T847','T857','T880', 'U04', 'M726', 'N49', 'U071', 'U072' |
| **5** | **Cancer** | 'C00', 'C01', 'C02', 'C03', 'C04', 'C05', 'C06', 'C07', 'C08', 'C09', 'C10', 'C11', 'C12', 'C13', 'C14', 'C15', 'C16', 'C17', 'C18', 'C19','C20', 'C21', 'C22', 'C23', 'C24', 'C25', 'C26', 'C27', 'C28', 'C29',           'C30', 'C31', 'C32', 'C33', 'C34', 'C35', 'C36', 'C37', 'C38', 'C39', 'C40', 'C41', 'C42', 'C43', 'C44', 'C45', 'C46', 'C47', 'C48', 'C49','C50', 'C51', 'C52', 'C53', 'C54', 'C55', 'C56', 'C57', 'C58', 'C59',            'C60', 'C61', 'C62', 'C63', 'C64', 'C65', 'C66', 'C67', 'C68', 'C69', 'C70', 'C71', 'C72', 'C73', 'C74', 'C75', 'C76', 'C77', 'C78', 'C79','C80', 'C81', 'C82', 'C83', 'C84', 'C85', 'C86', 'C87', 'C88', 'C89',           'C90', 'C91', 'C92', 'C93', 'C94', 'C95', 'C96', 'C97', 'D32', 'D33', 'D35', 'D42', 'D43', 'D44', 'D45', 'D46', 'D47' |
| **6** | **Diabetes** | 'E10', 'E11', 'E12', 'E13', 'E14' |
| **7** | **Cardiovascular** | 'G45', 'H34', 'I00', 'I01', 'I02', 'I03', 'I04', 'I05', 'I06', 'I07', 'I08', 'I09', 'I10', 'I11', 'I12', 'I13', 'I14', 'I15', 'I16', 'I17', 'I18', 'I19', 'I20', 'I21', 'I22', 'I23', 'I24', 'I25', 'I26', 'I27', 'I28', 'I29', 'I30',                    'I31', 'I32', 'I33', 'I34', 'I35', 'I36', 'I37', 'I38', 'I39', 'I40', 'I41', 'I42', 'I43', 'I44', 'I45', 'I46', 'I47', 'I48', 'I49', 'I50', 'I51', 'I52', 'I53', 'I54', 'I55', 'I56', 'I57', 'I58', 'I59', 'I60',                    'I61', 'I62', 'I63', 'I64', 'I65', 'I66', 'I67', 'I68', 'I69', 'I70', 'I71', 'I72', 'I73', 'I74', 'I75', 'I76', 'I77', 'I78', 'I79', 'I80', 'I81', '182', 'I83', 'I84', 'I85', 'I86', 'I87', 'I88', 'I89', 'I90', 'I91', 'I92', 'I93',                     'I94', 'I95', 'I96', 'I97', 'I98', 'I99' |
| **8** | **Lung** | 'J41', 'J42', 'J43', 'J44', 'J45', 'J46', 'J47', 'J84', 'J98' |
| **9** | **Dementia** | 'F00', 'F02', 'F03', 'G30', 'G31' |
| **10** | **Kidney** | 'N18' |
| **11** | **Liver** | 'K70', 'K72' |
| **12** | **Immune system** | 'D80', 'D81', 'D82', 'D83', 'D84', 'Z94' |

**Table F. Contaminant microbes:** List of microbes identified as contaminants

| **Contaminants** | 'BACILLUS CEREUS', 'STREPTOCOCCUS EQUI SSP EQUI', 'BACILLUS CIRCULANS', 'STREPTOCOCCUS EQUI SSP ZOOEPIDEMICUS', 'BACILLUS FIRMUS',​ 'STREPTOCOCCUS GORDONII', 'BACILLUS LICHENIFORMIS', 'STREPTOCOCCUS INTERMEDIUS', 'BACILLUS MEGATERIUM • STREPTOCOCCUS MITIS', 'BACILLUS PUMILUS', ​ 'STREPTOCOCCUS MITIS', 'STREPTOCOCCUS ORALIS', 'BACILLUS SPECIES', 'STREPTOCOCCUS MUTANS', 'BACILLUS SPHAERICUS', 'STREPTOCOCCUS PYOGENES', ​ 'BACILLUS SUBTILIS', 'STREPTOCOCCUS SALIVARIUS', 'COAGULASE NEGATIVE STAPHYLOCOCCUS', 'STREPTOCOCCUS SANGUINIS', 'CORYNEBACTERIUM JEIKEIUM',​ 'STREPTOCOCCUS VESTIBULARIS', 'CORYNEBACTERIUM SPECIES', 'STREPTOCOCCUS VIRIDANS GROUP', 'CORYNEBACTERIUM XEROSIS', 'MICROCOCCUS LUTEUS', ​ 'MICROCOCCUS LYLAE', 'MICROCOCCUS LUTEUS', 'STAPHYLOCOCCUS ARLETTAE','MICROCOCCUS SPECIES', 'STAPHYLOCOCCUS CAPRAE', 'STAPHYLOCOCCUS SPP', 'STAPHYLOCOCCUS CARNOSUS SSP CARNOSUS', 'STAPHYLOCOCCUS AUREUS', 'STAPHYLOCOCCUS GALLINARUM', 'STAPHYLOCOCCUS AURICULARIS', ​ 'STAPHYLOCOCCUS HOMINIS SSP HOMINIS','STAPHYLOCOCCUS CAPITIS', 'STREPTOCOCCUS ALACTOLYTICUS', 'STAPHYLOCOCCUS COHNII SSP COHNII', 'STREPTOCOCCUS CRISTATUS',​ 'STAPHYLOCOCCUS COHNII SSP UREALYTICUS',​ 'STREPTOCOCCUS CONSTELLATUS SSP CONSTELLATUS','STAPHYLOCOCCUS EPIDERMIDIS', 'STREPTOCOCCUS CONSTELLATUS SSP PHARYNGIS', 'STAPHYLOCOCCUS HAEMOLYTICUS', ​ 'STREPTOCOCCUS HYOINTESTINALIS', 'STAPHYLOCOCCUS HOMINIS', 'STREPTOCOCCUS MITIS/STREPTOCOCCUS ORALIS', 'STAPHYLOCOCCUS INTERMEDIUS', 'STREPTOCOCCUS PARASANGUINIS', 'STAPHYLOCOCCUS KLOOSII', 'STREPTOCOCCUS PLURANIMALIUM', 'STAPHYLOCOCCUS LENTUS', ​ 'STREPTOCOCCUS SOBRINUS', 'STAPHYLOCOCCUS LUGDUNENSIS','STREPTOCOCCUS THERMOPHILUS', 'STAPHYLOCOCCUS SACCHAROLYTICUS', 'STREPTOCOCCUS THORALTENSIS', 'STAPHYLOCOCCUS SAPROPHYTICUS', 'STREPTOCOCCUS SPP', 'STAPHYLOCOCCUS SCHLEIFERI', 'DIPHTHEROIDS SPP', 'STAPHYLOCOCCUS SCIURI', 'CORYNEBACTERIUM STRIATUM', 'STAPHYLOCOCCUS SIMULANS', 'NON HAEMOLYTIC STREPTOCOCCIS', 'STAPHYLOCOCCUS SPECIES', 'BABESIA SPP', 'STAPHYLOCOCCUS WARNERI', ​ 'CORYNEBACTERIUM MINUTISSMUM', 'STAPHYLOCOCCUS XYLOSUS','CORYNEBACTERIUM AMYCOLATUM', 'STREPTOCOCCUS AGALACTIAE', 'MICROMONAS MICROS',​ 'STREPTOCOCCUS ANGINOSUS',​ 'STAPHYLOCOCCUS PASTEURI', 'STREPTOCOCCUS CONSTELLATUS' |
| --- | --- |

**List A. Groups of various microbiology tests**

**'annet'**: ['ABSCESS', 'ABSCESS (TBA)', 'ABSCESS (VAB)', 'ACITES PÅ BL.K.FLASKE', 'AMPUTASJONSSTUMP', 'ANNET', 'ANNET (ANS)', 'ANNET (VAN)'],

**'anus'**: ['ANUSSEKRET', 'ANUSSEKRET (ANUM)', 'ANUSSEKRET (ANUP)', 'ANUSSEKRET (ANUS)', 'ANUSSEKRET (VANU)', 'ASCITES', 'ASPIRAT', 'ASPIRAT (VAS)', 'ASPIRAT PÅ BL.K.FLASKE', 'AUTOPSIMATRIALE', 'AUTOPSIMATRIALE (VAU)', 'AXILLE', 'AXILLE (MRSA)', 'BAKTERIESTAMME'],

**'bein'**: ['BEIN FRA BEINBANK', 'BEIN TIL BEINBANK', 'BEINMARG', 'BEINMARG (BEM)', 'BEINVEV'],

**'biopsi'**: ['BIHULESEKRET (BIH)', 'BIOPSI', 'BIOPSIMATERIALE', 'BIOPSIMATERIALE (TBI)', 'BIOPSIMATERIALE (VBI)'],

**'blod'**: ['BLOD - ISOLATOR', 'BLOD - ISOLATOR (TBLI)', 'BLODKULTUR (BLS)', 'BLODKULTUR (BLS1)'],

**'blood_culture_test': ['BLODKULTUR'],**

**'bronki'**: ['BRONKIALBØRSTE (VBR)', 'BRONKIALSKYLLEVÆSKE', 'BRONKIALSKYLLEVÆSKE (TBS)', 'BRONKIALSKYLLEVÆSKE (VBS)'],

**'melk'**: ['BRYSTMELK', 'BURSAVÆSKE', 'CERVIX (CERC)', 'CERVIX (CERM)', 'CERVIX-/URETHRASEKRET (CU', 'CERVIX-/VAGINALSEKRET', 'CERVIXSEKRET', 'CERVIXSEKRET (VCE)', 'CH-UROGENITALSEKRET', 'CH-UROGENITALSEKRET (UROM', 'CORNEAAVSKRAP', 'CYSTEINNHOLD', 'DIALYSAT PÅ BL.K.FLASKE', 'DIALYSEVÆSKE', 'DRENSPISS', 'DRENSVÆSKE', 'DRENSVÆSKE PÅ BL.K.FLASKE'],

**'edta'**: ['EDTA-BLOD', 'EDTA-BLOD (BEDT)', 'EDTA-BLOD (EDTA)', 'EDTA/UTSTRYK MALARIA', 'EJAKULAT (EJA)', 'EKSPEKTORAT', 'EKSPEKTORAT (EXS)', 'EKSPEKTORAT (TEX)', 'EKSPEKTORAT (VEK)', 'ELUAT', 'FISTEL', 'FOSTERVANN', 'FOSTERVANN (VFO)'],

**'faeces'**: ['FÆCES', 'FÆCES (FÆ)', 'FÆCES (FÆCB)', 'FÆCES (FÆCD)', 'FÆCES (FÆCP)', 'FÆCES (FÆD)', 'FÆCES (FÆFP)', 'FÆCES (FÆP)', 'FÆCES (VFÆ)', 'FÆCES (VFÆB)', 'FÆCES (VFÆN)', 'FÆCES (VFÆV)', 'GALLEVEISPRØVE', 'GENITALSEKRET (VGF)'],

**'hal'**: ['HALSSEKRET', 'HALSSEKRET (HALC)', 'HALSSEKRET (HALG)', 'HALSSEKRET (HALM)', 'HALSSEKRET (HAS)', 'HALSSEKRET (MRSA)', 'HALSSEKRET (VHA)', 'HALSSEKRET (VHAR)'],

**'hud'**: ['HUD', 'HUD (MRSA)', 'HUDAVSKRAP', 'HUDAVSKRAP (VHU)', 'HÅR', 'INDUSERT SPUTUM', 'INDUSERT SPUTUM (TSPU)', 'INNSTIKKSTED', 'KATETERSPISS', 'LARYNXSEKRET'],

**'led'**: ['LEDDVÆSKE', 'LEDDVÆSKE - ANRIKET', 'LGV (LYMFOGRAN. VENEREUM)', 'LYSAT', 'LYSKE (LYS)', 'MELK', 'MORSMELK'],

**'naso'**: ['MRSA REFERANSESTAMME (MRS', 'MUNNHULE', 'MUNNSEKRET', 'MUNNSEKRET (VMU)', 'NASOFARYNKS- OG HALSPRØVE', 'NASOPHARYNXASPIRAT (LUFA)', 'NASOPHARYNXASPIRAT (LUFT)', 'NASOPHARYNXASPIRAT (NAI)', 'NASOPHARYNXSEKRET', 'NASOPHARYNXSEKRET (NAS)', 'NASOPHARYNXSEKRET (VNAR)', 'NAVLESEKRET', 'NAVLESTRENG - BIT', 'NEGL', 'NEGL (NEGL)', 'NESESEKRET', 'NESESEKRET (DIA)', 'NESESEKRET (MRSA)'],

**'plasma'**: ['NONHUMANT MATERIALE', 'OPERASJONS-SÅR', 'OPPKAST', 'PACEMAKERTRÅD', 'PARAFININNSTØPT VEV', 'PD-DIALYSAT', 'PERICARDVÆSKE', 'PERICARDVÆSKE (VPEC)', 'PERINEUM', 'PERINEUM (MRSA)', 'PERITONEALVÆSKE', 'PERITONSILLÆRABSESS', 'PLACENTA', 'PLASMA', 'PLASMA (PLAS)', 'PLEURAV. PÅ BL.K.FLASKE', 'PLEURAVÆSKE', 'PLEURAVÆSKE (TPL)', 'PLEURAVÆSKE (VPLE)', 'PUSS (PSD)', 'PUSS/SEKRET', 'PUSS/SEKRET (TPU)', 'PUSS/SEKRET (VPUS)', 'RECTUMSEKRET (PREC)', 'SEKRET (SEK)', 'SERUM', 'SERUM (SSE)', 'SKYLLEVÆSKE (VSK)', 'SOPPKULTUR', 'SPINALVÆSKE', 'SPINALVÆSKE (VSP)', 'SPIRAL', 'SPISS AV CVK', 'SPUTUM (VSPU)', 'SÅRSEKRET', 'SÅRSEKRET (MRSA)', 'SÅRSEKRET (SÅS)', 'SÅRSEKRET (VSÅ)', 'TRACHEALASPIRAT', 'TRANSPLANTATMEDIUM', 'TRANSTRACHEALT ASPIRAT (T', 'TUBESEKRET (VTU)'],

**'tunge'**: ['TUNGESEKRET', 'TUNGESEKRET (TUNS)', 'TUNGESEKRET (VTUN)', 'TÅREVÆSKE'],

**'urin'**: ['URETHRA', 'URETHRA (UREM)', 'URETHRASEKRET', 'URETHRASEKRET (VUR)', 'URIN', 'URIN (CHLAMYDIA)', 'URIN (CHU)', 'URIN (CLUM)', 'URIN (CLUR)', 'URIN (TUR)', 'URIN (URS)', 'URIN (VURI)', 'URIN BLÆREPUNKSJON', 'URIN/TRANSPORTAGAR (URC)', 'UROGENITALSEKRET', 'UROGENITALSEKRET (URO)', 'USPESIFISERT (USP)', 'USPESIFISERT (VUS)', 'USPESIFISERT(MRSA)', 'UTERUSSEKRET (PUTE)', 'UTSTRYK', 'VAGINA', 'VAGINA (VAGM)', 'VAGINALPENSEL/URIN', 'VAGINALSEKRET', 'VAGINALSEKRET (CHP)', 'VAGINALSEKRET (VVG)', 'VESIKKELINNHOLD', 'VESIKKELINNHOLD (VESB)', 'VEV (TVV)', 'VEV/BIOPSI', 'VEV/BIOPSI (BIO)', 'VULVA (VVU)'],

**'ear'**: ['ØRESEKRET', 'ØRESEKRET (ØRS)', 'ØRESEKRET, HØYRE ØRE', 'ØRESEKRET, VENSTRE ØRE'],

**'eye'**: ['ØYEKAMMERVÆSKE', 'ØYESEKRET', 'ØYESEKRET (VØY)', 'ØYESEKRET (ØYC)', 'ØYESEKRET (ØYM)', 'ØYESEKRET - HØYRE ØYE', 'ØYESEKRET - HØYRE ØYE (VØ', 'ØYESEKRET - VENSTRE ØYE', 'ØYESEKRET - VENSTRE ØYE (' ]}
