# Supplementary material for: Leveraging explainable artificial intelligence for early prediction of bloodstream infections using historical electronic health records
Source: PLOS Digit Health. 2024 Nov 14;3(11):e0000506. doi: 10.1371/journal.pdig.0000506 (PMC11563427; doi:10.1371/journal.pdig.0000506)
Supplement: Attachment — Submitted filename: Response_to_Reviewers.docx [file pdig.0000506.s003.docx]

Responses To Reviewer’s Comments

**Additional Editor Comments:**

I share a similar question as the reviewer regarding the model's performance. Also, the speaker should examine the interpretation of the SHAP values. For example, a lower SHAP value represents a negative influence on the prediction rather than indicating that the predictor is not important (e.g., 'anomaly_score').

*Response:* We appreciate the editor's insight into the interpretation of SHAP values. We understand that SHAP values provide a measure of the contribution of each feature to the model's output, where a positive SHAP value indicates a feature that contributes positively to the prediction, and a negative SHAP value indicates a feature that contributes negatively. In our analysis, we have carefully examined the SHAP values across all features, including 'anomaly_score'. We have removed the incorrect sentence on interpretation of ‘anomaly_score’ SHAP values and clarified that a lower SHAP value for 'anomaly_score' does not imply a lack of importance at the end of Results Section 2.4.

**1. Reviewer 1’s Comments**

Table 1 gives replies to each comment by the reviewer 1.

**Table 1 is a tool that organizes our replies to each comment by Reviewer 1, making it easier for the reader to locate specific responses.**

| **S/No.** | **Comments** | **Responses** |
| --- | --- | --- |
| 1 | [Case Study] To further understand the proposed method, the authors may provide a case study. | Thank you for the constructive comment, we agree that a more focused example can help illustrate the practical application and effectiveness of our approach. We have included the case study involving a balanced subset of 1,000 patients from our dataset. The case study highlights how our framework utilizes administratively stored historical medical data to derive meaningful features to train static ML models and create sequences to train sequential and SOTA ML models. |
| 2 | [Motivation and Contribution] As known, there are some existing relevant methods in the community. The authors should include these studies in the manuscript. What are the improvements of the proposed method compared with these existing methods? What problems did the previous works exist? How to solve these problems? The authors may consider analyzing the problems of the previous works and how to address these problems in the manuscript. Please explain that. | We thank the reviewer for their valuable suggestion. We have now expanded our literature review to include a detailed subsection (Section 3.4: Data Challenges and Strategies) that discusses the problems identified in previous works, particularly related to data imbalance, feature selection, and model interpretability. We also outline how our study addresses these issues.  **Data Imbalance:** Many previous studies struggled with the challenge of imbalanced datasets, leading to biased models that were less effective at identifying true positive cases of BSI. To address this, we applied class weights and performed a case study on a balanced dataset. These strategies improved the model's sensitivity and overall performance, particularly in identifying minority class cases, which are crucial in medical diagnosis.  **Feature Selection:** Our study emphasizes the importance of deriving meaningful features from raw data. We extracted relevant features from the historical EHRs that are predictive of BSI, a step that distinguishes our work from many prior studies that relied heavily on predefined or less comprehensive feature sets. This feature engineering approach significantly enhances the predictive power of our models.  **Interpretability:** A major limitation of previous models has been their lack of transparency, making them difficult to integrate into clinical practice. We addressed this by employing SHAP values, which provide clear and interpretable insights into how each feature influences the model's predictions. This level of transparency is essential for gaining the trust of healthcare professionals and facilitating the adoption of AI-based tools in clinical settings.  **Comparison of Data Modeling Techniques:** Our study is unique in its comparison of two distinct data modeling approaches—sequential and static. We demonstrated that while static models generally outperform sequential ones, both approaches have their strengths, particularly when combined. This dual approach allows for a more comprehensive analysis and understanding of the data, leading to better-informed clinical decisions.  These improvements and solutions to the problems identified in previous studies have been thoroughly discussed in the revised manuscript Discussion Section. We believe that these contributions significantly advance the field and provide a solid foundation for future research in BSI prediction. |
| 3 | [SOTA] The authors should compare the proposed method with some recent algorithms. | We appreciate the reviewer’s suggestion to compare our proposed method with recent SOTA algorithms. In response to this comment, we have included a detailed comparison of our method against the recent suggested SOTA ML models, including the Densely Knowledge-aware Network (DKN) and CapMatch models.  To address this comment, we have:  - Incorporated SOTA Models in Comparative Analysis in the main and case study.  - Added text in the Discussion section first paragraph highlighting the comparison wth SOTA models. |
| 4 | [Mathematics] The authors consider some mathematical reasonings in the manuscript. | Thank you for your comment. We have included details on the mathematical formulations for the ML models and performance metrics employed in the Supplementary Information File 1, Section 1.1-1.3. We have cross-refernced it in the main manuscript in the Method and Materials Section. |
| 5 | [Research Gap] The research gaps should be highlighted and explained in the manuscript. | We appreciate the reviewer’s focus on the importance of highlighting and addressing research gaps. In response to this insightful feedback, we have revised the manuscript to more explicitly identify and explain the research gaps in the context of BSI prediction models and to articulate how our innovative approach addresses these gaps. Specifically, we have:  **Enhanced the Literature Review**: We expanded the literature review to clearly identify the critical gaps in existing BSI prediction models, including issues related to data imbalance, feature selection, and model interpretability. These gaps are now discussed in detail in the revised Discussion section.  **Illustrated Our Contributions**: In the Discussion Section 3.3 and 3.4. We have articulated how our study not only addresses these gaps but also introduces novel methodologies that enhance the predictive power and clinical applicability of BSI prediction models. This includes the use of class weighting, comprehensive feature engineering from EHRs, and the application of SHAP values for improved model transparency.  **Provided Contextual Comparison**: In the Discussion Section 3.2. We have contextualized our contributions by comparing our approach to existing methods, demonstrating the advancements our study offers in addressing these critical gaps. |
| 6 | [State Similar Studies] There are some existing algorithms that have some merits similar to the proposed method. The authors should explain the differences between the proposed method and existing algorithms, including Densely Knowledge-aware Network for Multivariate Time Series Classification, CapMatch: Semi-supervised Contrastive Transformer Capsule with Feature-based Knowledge Distillation for Human Activity Recognition, DTCM: Deep Transformer Capsule Mutual Distillation for Multivariate Time Series Classification, and Deep Contrastive Representation Learning With Self-Distillation. | We appreciate the reviewer's insightful comment. To address this, we have provided a detailed comparison between our proposed framework and the mentioned models in the revised manuscript. Here is a summary of the key differences and the unique contributions of our approach:  **Objective and Application Domain:**  The DKN and CapMatch are primarily designed for tasks such as news recommendation, human activity recognition, and general multivariate time series classification. While these tasks share commonalities with our objective, they are not directly targeted at clinical prediction tasks such as BSI prediction, where the temporal dynamics and feature engineering from EHRs can be crucial.  Our framework is specifically tailored for BSI prediction, focusing on leveraging administratively stored historical medical data and addressing domain-specific challenges such as data imbalance, feature selection, and model interpretability, which are essential in clinical decision-making contexts.  **Feature Engineering and Domain Knowledge Integration:**  Unlike DKN and CapMatch, which integrate domain knowledge through external knowledge graphs or contrastive learning, our framework emphasizes deriving meaningful features directly from historical EHRs. This step is critical for clinical prediction, where the relevance and quality of input features significantly impact model performance and interpretability.  **Interpretability:** A significant limitation of the models mentioned is their lack of built-in interpretability mechanisms, which can hinder their adoption in clinical settings. Our framework addresses this by incorporating SHAP values, providing clear insights into how each feature influences the model’s predictions. This is essential for gaining trust among healthcare professionals.  **Performance Evaluation in Clinical Context:**  We have included a detailed comparison of our method against these SOTA models in both the main analysis and the case study section of our manuscript. While the results from DKN are promising, our framework has shown superior performance in BSI prediction, particularly in handling imbalanced datasets and delivering interpretable results. |
| 7 | [Computational Complexity] Computational complexity should be analyzed and compared in the manuscript. | Thank you for your comment. We have included details on the computational complexity for the various ML models analysing and comparing tem in the Materials and Methods Section 4.7. |

**2. Replies to reviewer 2’s comments**

Table 2 gives replies to each comment by the reviewer 2.

**Table 1 is a helpful tool that organizes our replies to each comment by Reviewer 2, making it easier for the reader to locate specific responses.**

| **S/No.** | **Comments** | **Replies** |
| --- | --- | --- |
| 1 | In line 246, does it indicate that the “Temporal ML models” utilizing additional time variable compared to “static models”? If so, is there explanation about why static model works better than the sequential model incorporating additional information. | We appreciate the reviewer's insightful comment and would like to clarify this point. The term "Temporal ML models" now changed to “Sequential ML models” for better clarity, indeed refers to models that incorporate an additional time dimension or sequence information to account for temporal dependencies in the data, as compared to "static models" that do not consider this temporal aspect. For the static models we have derived temporal features like ‘time_to_last’ episode and ‘total_los’ (cumulative LOS till the BC) to capture these temporal dependencies.  In our study, while we hypothesized that the temporal models (such as LSTM and GRU) would outperform static models due to their ability to capture time-dependent patterns, the results indicated that static models such as XGBoost and LightGBM actually performed better overall. This outcome can be attributed to several factors:  **Data Characteristics:** The temporal patterns in our dataset may not have been as strong or consistent as initially expected, leading to the static models, which focus on optimizing feature importance without considering sequence, outperforming the sequential models.  **Model Complexity:** Sequential models, while powerful, often require larger amounts of data to generalize well, especially when the patterns are subtle. The relatively smaller size of our dataset might have limited the performance of the temporal models.  **Feature Engineering:** The static models benefited significantly from comprehensive feature engineering that distilled essential predictive features from the data. These features likely encapsulated much of the temporal information in a form that static models could effectively leverage.  We have expanded our discussion on this topic in Section 3.1 of the Discussion, where we detail these factors and their potential impact on the observed model performances. Additionally, we have emphasized the importance of further research to explore the specific conditions under which temporal models might outperform static models in BSI prediction. |
| 2 | In the model performance section, a bunch of the model evaluations were used, is there a preferred metric here for bloodstream infection prediction? Especially given the fact of the imbalance feature of the dataset? Have you tried to apply your models on the balanced dataset and see if the model performance/SHAP changed? | Thank you for your insightful comment. We acknowledge the importance of choosing appropriate metrics when evaluating models trained on imbalanced datasets, such as those used for BSI prediction. In our study, we considered multiple performance metrics, each offering a different perspective on the model’s effectiveness, particularly in the context of imbalanced data.  **Preferred Metrics:**  **Area Under the Precision-Recall Curve (AUPRC):** Given the imbalanced nature of the dataset, where the number of positive cases BSIs is much smaller than the number of negatives, AUPRC was selected as a primary metric. AUPRC is particularly useful in this scenario as it focuses on the performance of the model in distinguishing between the positive class (BSI) and the negative class, without being overly influenced by the abundance of negative cases, unlike AUROC.  **F1-score:** This metric balances precision and recall, making it suitable for assessing performance in imbalanced datasets. It ensures that neither false positives nor false negatives are disproportionately favored in the evaluation process.  **AUROC (Area Under the Receiver Operating Characteristic Curve):** While AUPRC was prioritized, AUROC was also reported for completeness. However, as AUROC can be less informative for highly imbalanced datasets, we emphasize that AUPRC and F1-score are more indicative of model performance in this context.  **Handling Imbalanced Data:**  **Class Weights:** In the initial model training, class weights were applied to mitigate the effect of class imbalance. This approach adjusted the cost function to penalize misclassifications of the minority class (BSI cases) more heavily, thereby encouraging the model to focus on correctly identifying these cases.  **Balanced Dataset Analysis:** In response to reviewer 1 comment 1’s suggestion, we conducted case study using a balanced subset of our dataset. We observed the following:  **Performance Metrics:** The overall performance metrics, particularly AUPRC and F1-score, showed an improvement in sensitivity to the minority class. This was expected as balancing the dataset inherently reduces the disparity between precision and recall.  **SHAP Value Analysis:** The SHAP value distributions and the relative importance of features remained consistent with our original findings, indicating that the model's interpretability and feature contributions were robust to the dataset’s class distribution.  The metrics chosen in our original analysis (AUPRC, F1-score, and AUROC) were selected to address the challenges posed by an imbalanced dataset. After testing on a balanced subset, we confirmed that the overall conclusions regarding model performance and feature importance remained stable, reinforcing the validity of our approach. |
| 3 | In Table 2, I am surprised that the precision of RF is 0.8510, which surpass other models including other tree based models, but a low recall of 0.2201; Similar situation applies for NN but not as extreme as RF. Just wonder if it is still affected by the unbalance nature of the dataset, might be good to run a sensitivity analysis through adjusting the weights of the minority group (or resampling), which could potentially make precision/recall score more stable. Maybe Ensemble technique could be used to provide an overall performance. | We appreciate the reviewer's insightful observation regarding the discrepancy between the precision and recall metrics for the RF and NN models. This disparity indeed highlights the challenges associated with class imbalance in the dataset, which can lead to a model that is overly precise but not sufficiently sensitive to the minority class.  In response to this comment, we have performed additional analyses to investigate the impact of class imbalance on the model's performance metrics. Specifically, we employed class weighting as a strategy to address the imbalance, adjusting the weights inversely proportional to class frequencies in the training data. Additionally, we explored resampling techniques, including both oversampling of the minority class and undersampling of the majority class, to further assess their impact on the model's precision and recall.  Our findings, now incorporated into the revised manuscript, indicate that applying class weights indeed improves the recall of the RF model, bringing it closer to a balanced trade-off between precision and recall. Specifically, we observed that while precision slightly decreased, recall improved significantly, resulting in a more balanced F1-score, as shown in the updated Table 2 of the manuscript.  Furthermore, we explored the use of ensemble techniques such as bagging and boosting to further stabilize the model's performance. The ensemble methods, particularly XGBoost and LightGBM, demonstrated more stable precision and recall metrics compared to the standalone RF model. These findings suggest that ensemble methods are more robust to class imbalance and are thus recommended for similar predictive tasks in future studies.  To summarize, the following changes have been made to the manuscript:  **Inclusion of Class Weighting and Resampling(case study) Results:**  The updated Table 2 reflects the performance metrics after applying class weighting and resampling strategies, highlighting the improvements in recall for the RF model.  We believe these additional analyses address the reviewer's concerns and enhance the robustness of our findings. |
| 4 | Since Supplementary Table 3 shows some features are highly correlated, it would be great to explain whether/to what extend it affect the shap value, so that the independence assumption are still met. | Thank you for this insightful comment. We understand the concern regarding the potential impact of multicollinearity on SHAP values, as SHAP relies on the assumption of feature independence when attributing importance to each feature.  **Impact of Multicollinearity on SHAP Values:** Multicollinearity can indeed influence SHAP values by making it difficult to isolate the contribution of each feature. When features are highly correlated, SHAP values might allocate similar importance to both features, potentially inflating or distorting their individual contributions to the model's predictions. This can result in less reliable interpretations of feature importance, as SHAP values might reflect shared information between correlated features rather than distinct contributions.  **Steps Taken to Mitigate the Impact:**  **Correlation Analysis and Feature Selection:**  Before model training, we performed a thorough correlation analysis, as documented in Supplementary Table 3. Highly correlated features were identified.  **Interpretation of SHAP Values:**  In the context of correlated features, we have taken care to interpret SHAP values with caution, acknowledging the possibility of shared information between features.  **Clarifications in the Revised Manuscript:** We have added a detailed explanation of the potential impact of feature correlation on SHAP values and the steps we took to address this concern in Section 2.5. Additionally, we cross-referenced this discussion with the correlation analysis presented in Supplementary Table 3. |
| 5 | The current SHAP value is from XGBoost model, is it because it has the “balanced performance” (indicated by line 370)? Just wonder if it is possible to have overall “feature importance” ignoring which particular model is picked. | We appreciate the reviewer’s insightful question regarding the SHAP values and the selection of the XGBoost model. In our study, we selected the XGBoost model for generating SHAP values due to its balanced performance across various metrics, including AUROC, precision, recall, and F1-score, as detailed in Section 2.2 of the manuscript. The balanced performance of XGBoost makes it an ideal candidate for deriving SHAP values because it provides a comprehensive view of feature importance that is not overly biased by any particular metric.  Regarding the possibility of generating an overall “feature importance” that is independent of the model, this would indeed be an ideal scenario. However, SHAP values are model-specific because they explain the output of a particular model by assigning each feature an importance value that reflects its contribution to the prediction made by that model. Therefore, the SHAP values generated from different models, such as LightGBM, RF, or NN would likely differ because each model might use the features differently based on its architecture and training.  To provide a more comprehensive analysis, we could aggregate SHAP values across multiple models by calculating the mean SHAP value for each feature across all models. This approach would offer a more generalized view of feature importance, reducing model-specific biases. However, this method also risks diluting the interpretability of SHAP values, which are inherently designed to explain individual model decisions.  In the revised manuscript, we have expanded our discussion in Section 3.5 to include these considerations and have mentioned the potential for future work to explore aggregate SHAP values across different models to achieve a more generalized feature importance measure. This approach, while complex, could provide valuable insights and we believe it could be an interesting direction for future research |
| 6 | Overall for the XBSI framework, in Figure 1, due to the performance for difference model type dynamic/static, does it suggest static models are suitable with the prediction task for the future. | Thank you for your insightful comment regarding the suitability of static models for future prediction tasks within the XBSI framework. We acknowledge that our study revealed that static models, such as XGBoost and LightGBM, demonstrated superior overall performance in comparison to sequential models like LSTM and GRU. This observation is indeed significant and merits a deeper discussion, which we have now expanded in the revised manuscript (see Discussion Section 3.1).  **Key Considerations and Findings:**  **Data Characteristics and Temporal Dependencies:** The dataset's characteristics, particularly the temporal patterns, were not as strong or consistent as initially hypothesized. This could explain why the static models, which do not inherently account for sequence or time-dependent patterns, outperformed sequential models in this specific context. The static models benefited greatly from the feature engineering process, where temporal aspects were distilled into features like ‘time_to_last’ episode and ‘total_los’ (cumulative LOS till the BC). These features captured relevant temporal information effectively, enabling the static models to utilize them without the complexity of sequence modeling.  **Model Complexity and Data Volume:** Sequential models, such as LSTM and GRU, are powerful in capturing temporal dependencies but generally require large volumes of data to train effectively. Given the relatively smaller size of our dataset, the complexity of these models may have led to overfitting or underperformance. In contrast, the static models, which rely on simpler assumptions about the data, were able to generalize better under these conditions.  **Interpretability and Clinical Applicability:** The static models also provided clearer and more interpretable results, which is a critical consideration in clinical settings. The SHAP value analysis conducted on the XGBoost model, for example, offered insights into feature importance that were straightforward for clinical practitioners to understand and act upon. This level of interpretability is essential for integrating AI-based models into healthcare decision-making processes.  **Future Research Directions:** While the findings suggest that static models are well-suited for the current prediction task, it is important to note that this may not universally apply to all clinical prediction tasks or datasets. Future research could explore conditions under which sequential models might outperform static ones, particularly in larger datasets or where temporal patterns are more pronounced. Additionally, hybrid approaches that combine the strengths of both static and sequential models could offer a balanced solution, leveraging the interpretability of static models and the temporal awareness of sequential models.  In summary, while our results favor static models for the task at hand, we recommend a context-dependent approach when selecting model types for future prediction tasks. We have updated the manuscript to reflect this nuanced understanding and to suggest avenues for future exploration. |
